# Supplementary material for: Enantioselective Synthesis of the Active Sex Pheromone Components of the Female Lichen Moth, Lyclene dharma dharma, and Their Enantiomers
Source: Molecules. 2024 Jun 19;29(12):2918. doi: 10.3390/molecules29122918 (PMC11207095; doi:10.3390/molecules29122918)
Supplement: Supplementary file 1 [file molecules-29-02918-s001.zip › molecules-3042523-supplementary.pdf]

SUPPORTING INFORMATION

Enantioselective Synthesis of the Active Sex Pheromone Components of the Female Lichen Moth, *Lyclene dharma dharma*, and Their Enantiomers

Yun Zhou [1], Jianan Wang [2], Yueru Zhang[1], Xiaochen Fu [1], Hongqing Xie[1], Jinlong Han [1], Jianhua Zhang[1], Jiangchun Zhong[2], Chenggang Shan[1]*

[1] Institute of Industrial Crops, Shandong Academy of Agricultural Sciences, Jinan 250100, China.

[2] Department of Applied Chemistry, China Agricultural University, Beijing 100193, China.

* Correspondence: shanchenggang@126.com; Tel.: +86-053166655005

Table of Contents

S1. General information……………………………………………………………………….....2

S2. Synthesis of Mosher esters……………….…………………….…………………………………2

S3.^{1}H, ^{13}C spectra of the products……………………………………………......................................4

S4. Reference………………………………………..………..30

S1. General information

All reactions were conducted under an inert argon atmosphere within a Schlenk line system. Commercially available reagents were used as received, while solvents were distilled prior to use according to standard procedures. ^{1}HNMR spectra (500 MHz, TMS at δ 0.00 ppm or $CDCl_3$ at δ 7.26 ppm) and ^{13}CNMR spectra (126 MHz, $CDCl_3$ at δ 77.16 ppm as internal standard) were recorded on a Bruker DP-X500 spectrometer (Bruker Corporation, Beijing, China). High-resolution mass spectra (HRMS) were recorded on a Waters LCT Premier™ (Waters Corporation, Beijing, China) equipped with an electrospray ionization (ESI) mass spectrometer. Optical rotations were recorded on a Rudolph Research Analytical AUTOPOL-IV polarimeter (Rudolph Research Analytical, Beijing, China). Melting points were measured by a Stuart SMP3 Melt-Temp apparatus (Stuart Equipment, Beijing, China) and are reported uncorrected.

S2. Synthesis of Mosher esters

Scheme S1. Synthesis of Mosher esters **18-21**.

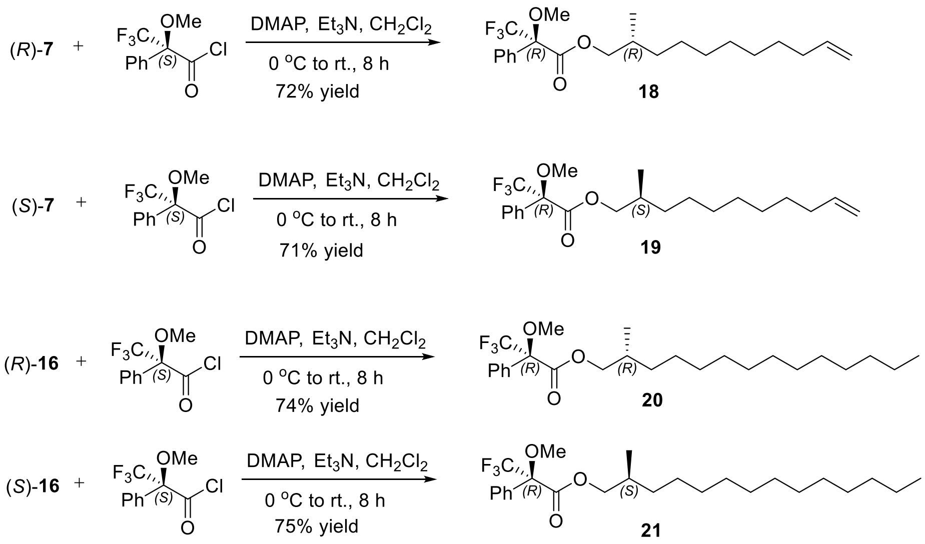

*(R)-2-methylundec-10-en-1-yl (R)-3,3,3-trifluoro-2-methoxy-2-phenylpropanoate (**18**)*

Under an argon atmosphere, (*R*)-2-methylundec-10-en-1-ol ((*R*)-**7**) (18.4 mg, 0.1 mmol, 1.0 equiv.) was added slowly to the solution of DMAP (12.2 mg, 0.1 mmol, 1.0 equiv.) and triethylamine (50.6 mg, 0.5 mmol, 5.0 equiv.) in dichloromethane (4 mL) at 0 °C. Subsequently, (*S*)-(-)-α-Methoxy-α-(trifluoromethy1) phenylacetyl chloride (50.6 mg, 0.2 mmol, 2.0 equiv.) was then added, causing the reaction mixture to turn yellow. The mixture was allowed to warm to room temperature and stirred continuously for overnight. The reaction was then quenched by adding 3 mL of water, followed by separation of the organic phase. The aqueous phase was extracted with DCM (3 × 10 mL). The combined organic layers were washed with brine (10 mL), dried over anhydrous Na_2SO_4, and concentrated under reduced pressure to yield the crude product. This product was purified by thin-layer chromatography to afford (*R*)-2-methylundec-10-en-1-yl (*R*)-3,3,3-trifluoro-2-methoxy-2-phenylpropanoate (**18**) (28.8 mg, 72% yield) as a colorless oil [1]. ^{1}H NMR (500 MHz, Chloroform-*d*) δ 7.53 – 7.51 (m, 2H), 7.41 – 7.39 (m, 3H), 5.85 – 5.78 (m, 1H), 5.03 – 4.90 (m, 2H), 4.23 (dd, *J* = 10.7, 5.6 Hz, 1H), 4.07 (dd, *J* = 10.7, 6.7 Hz, 1H),

3.56 (s, 3H), 2.06 – 2.01 (m, 2H), 1.87 – 1.81 (m, 1H), 1.40 – 1.26 (m, 11H), 1.18 – 1.12 (m, 1H), 0.91 (d, *J* = 6.8 Hz, 3H). HRMS (ESI, *m/z*): calculated for $[M + Na]^+$ $C_{22}H_{31}F_3O_3Na$ 423.2118, found: 423.2101.

*(S)-2-methylundec-10-en-1-yl (R)-3,3,3-trifluoro-2-methoxy-2-phenylpropanoate (**19**)*

Following the procedure previously described for the synthesis of **18**, (*S*)-2-methylundec-10-en-1-ol (*S*)-**7** (18.4 mg, 0.1 mmol, 1.0 equiv.) and (*S*)-(-)-α-Methoxy-α-(trifluoromethy1) phenylacetyl chloride (50.6 mg, 0.2 mmol, 2.0 equiv.) were reacted to give (*S*)-2-methylundec-10-en-1-yl (*R*)-3,3,3-trifluoro-2-methoxy-2-phenylpropanoate (**19**) (28.4 mg, 71% yield) as a colorless oil. ^{1}H NMR (500 MHz, Chloroform-*d*) δ 7.53 – 7.51 (m, 2H), 7.42 – 7.39 (m, 3H), 5.85 – 5.77 (m, 1H), 5.04 – 4.90 (m, 2H), 4.18 – 4.12 (m, 2H), 3.55 (s, 3H), 2.06 – 2.01 (m, 2H), 1.86 – 1.82 (m, 1H), 1.40 – 1.23 (m, 11H), 1.17 – 1.11 (m, 1H), 0.92 (d, *J* = 6.8 Hz, 3H). HRMS (ESI, *m/z*): calculated for $[M + Na]^+$ $C_{22}H_{31}F_3O_3Na$ 423.2118, found: 423.2102.

*(R)-2-methyltetradecyl (R)-3,3,3-trifluoro-2-methoxy-2-phenylpropanoate (**20**)*

Following the procedure previously described for the synthesis of **18**, (*R*)-2-methyltetradecan-1-ol ((*R*)-**16**) (22.8 mg, 0.1 mmol, 1.0 equiv.) and (*S*)-(-)-α-Methoxy-α-(trifluoromethy1) phenylacetyl chloride (50.6 mg, 0.2 mmol, 2.0 equiv.) were reacted to give (*R*)-2-methyltetradecyl (*R*)-3,3,3-trifluoro-2-methoxy-2-phenylpropanoate (**20**) (32.9 mg, 74% yield) as a colorless oil. ^{1}H NMR (500 MHz, Chloroform-*d*) δ 7.53 – 7.51 (m, 2H), 7.42 – 7.39 (m, 3H), 4.23 (dd, *J* = 10.7, 5.6 Hz, 1H), 4.07 (dd, *J* = 10.7, 6.7 Hz, 1H), 3.56 (s, 3H), 1.87-1.81 (m, 1H), 1.36 – 1.26 (m, 22H), 0.92 – 0.86 (m, 6H). HRMS (ESI, *m/z*): calculated for $[M + K]^+$ $C_{25}H_{39}F_3O_3K$ 483.2483, found: 483.2477.

*(S)-2-methyltetradecyl (R)-3,3,3-trifluoro-2-methoxy-2-phenylpropanoate (**21**)*

Following the procedure previously described for the synthesis of **18**, (*S*)-2-methyltetradecan-1-ol ((*S*)-**16**) (22.8 mg, 0.1 mmol, 1.0 equiv.) and (*S*)-(-)-α-Methoxy-α-(trifluoromethy1) phenylacetyl chloride (50.6 mg, 0.2 mmol, 2.0 equiv.) were reacted to give (*S*)-2-methyltetradecyl (*R*)-3,3,3-trifluoro-2-methoxy-2-phenylpropanoate (**21**) (33.3 mg, 75% yield) as a colorless oil. ^{1}H NMR (500 MHz, Chloroform-*d*) δ 7.53 – 7.51 (m, 2H), 7.42 – 7.38 (m, 3H), 4.16 – 4.15 (m, 2H), 3.55 (s, 3H), 1.86 – 1.82 (m, 1H), 1.35 – 1.26 (m, 21H), 1.15 – 1.10 (m, 1H), 0.91 (d, *J* = 6.7 Hz, 3H), 0.88 (t, *J* = 6.9 Hz, 3H). HRMS (ESI, *m/z*): calculated for $[M + K]^+$ $C_{25}H_{39}F_3O_3K$ 483.2483, found: 483.2484.

S3. ^{1}H and ^{13}C spectra of the products

Figure S1. ^{1}H NMR Spectrum of compound (*R*)-**5** (500 MHz, $CDCl_3$)

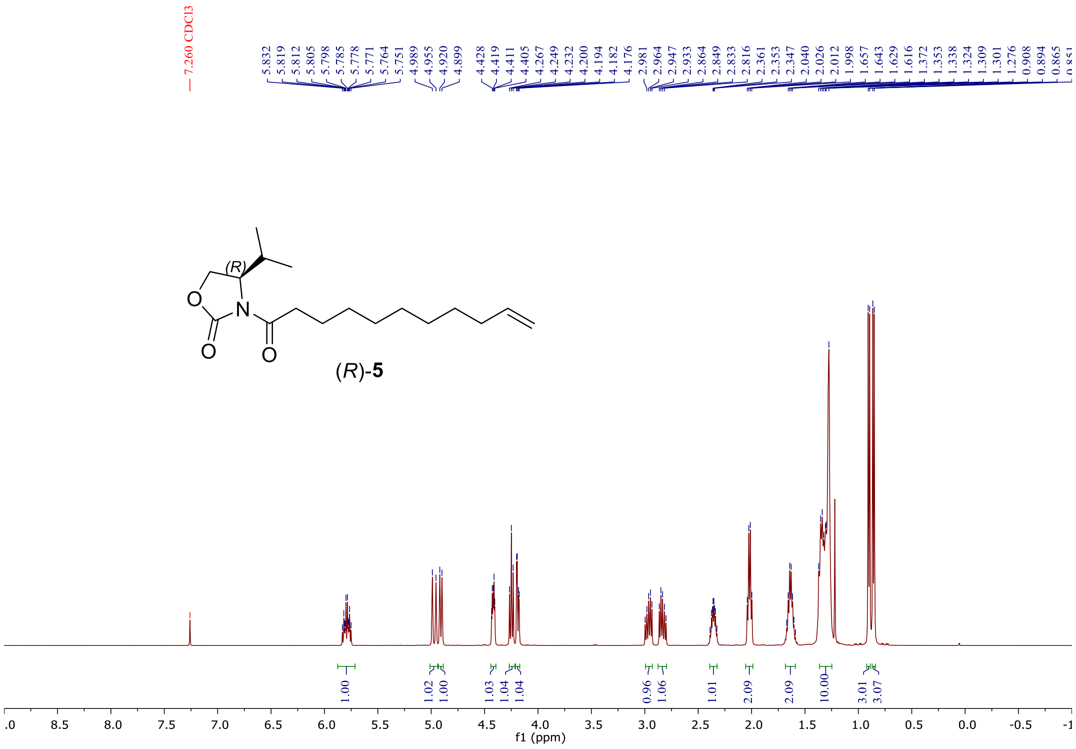

Figure S2. ^{13}C NMR Spectrum of compound (*R*)-**5** (125 MHz, $CDCl_3$)

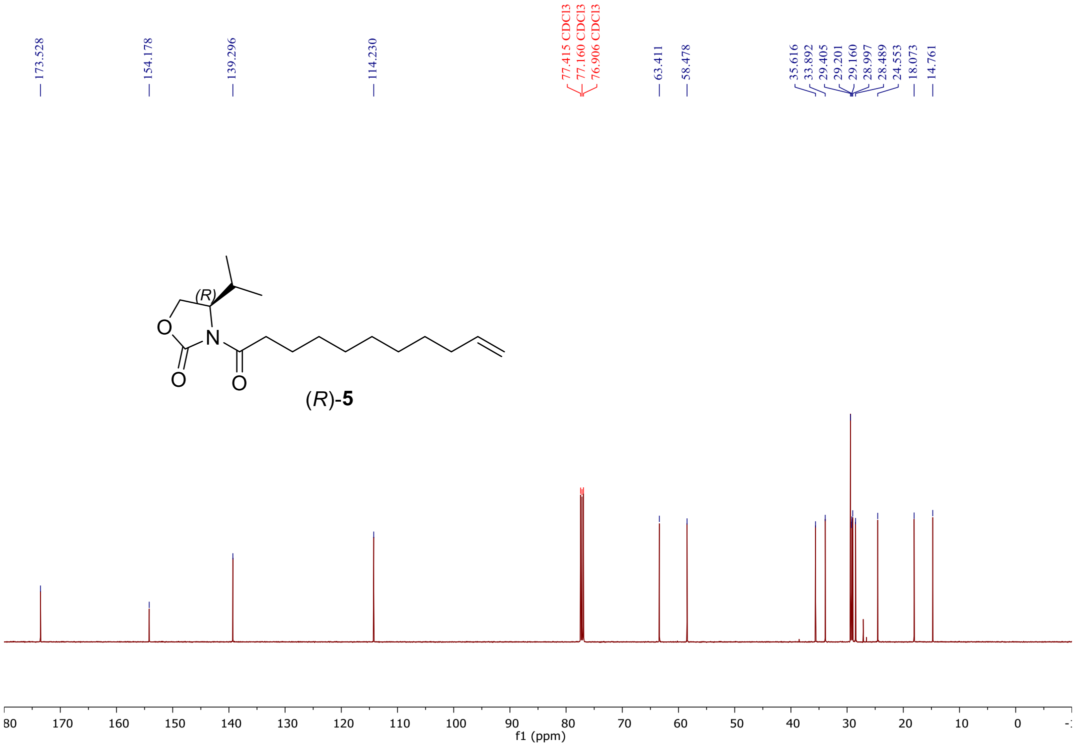

Figure S3. ¹H NMR Spectrum of compound (*R*, *R*)-**6** (500 MHz, $CDCl_3$)

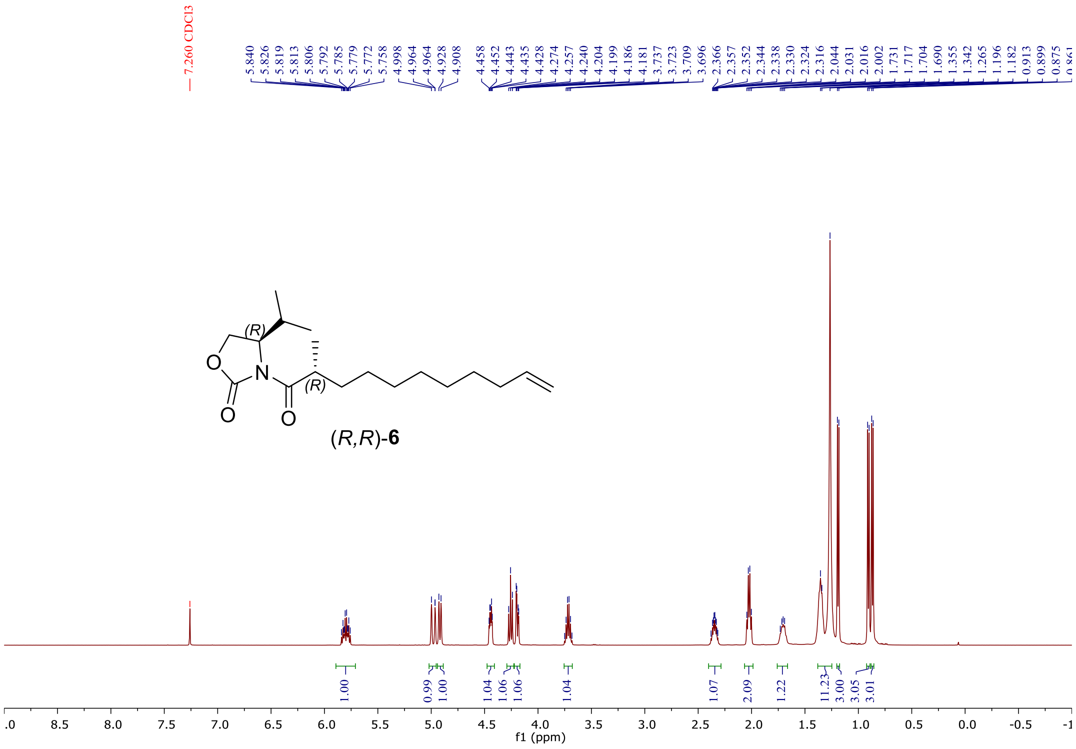

Figure S4. ¹³C NMR Spectrum of compound (*R*, *R*)-**6** (125 MHz, $CDCl_3$)

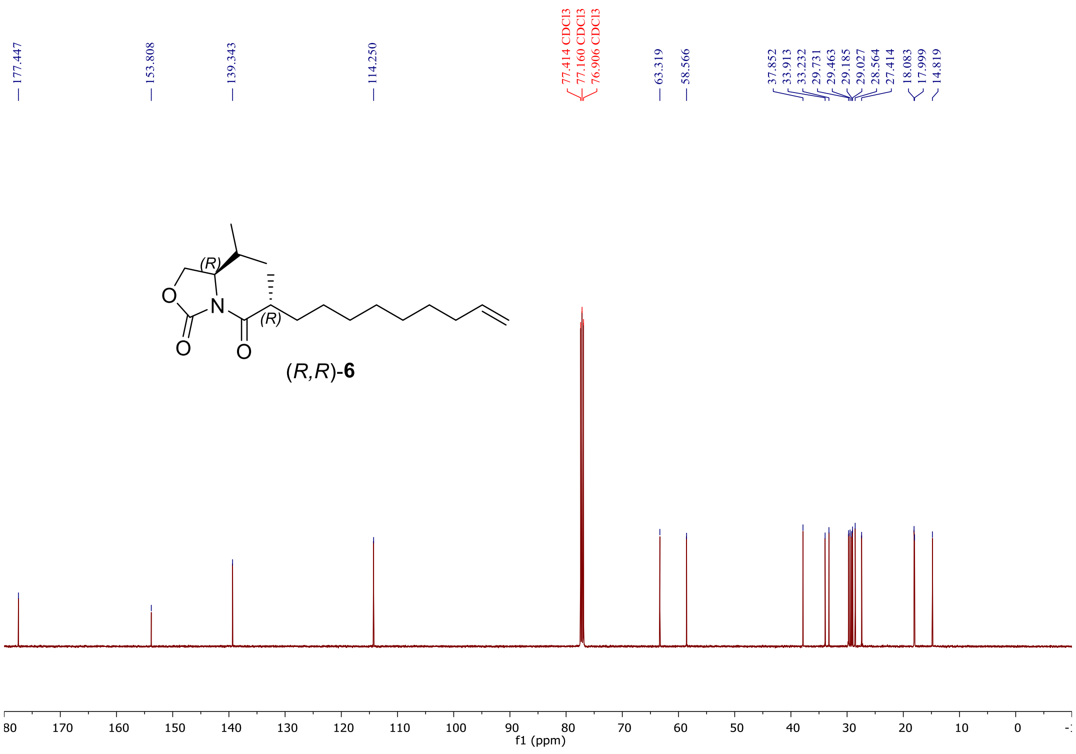

Figure S5. ^{1}H NMR Spectrum of compound (*R*)-**7** (500 MHz, $CDCl_3$)

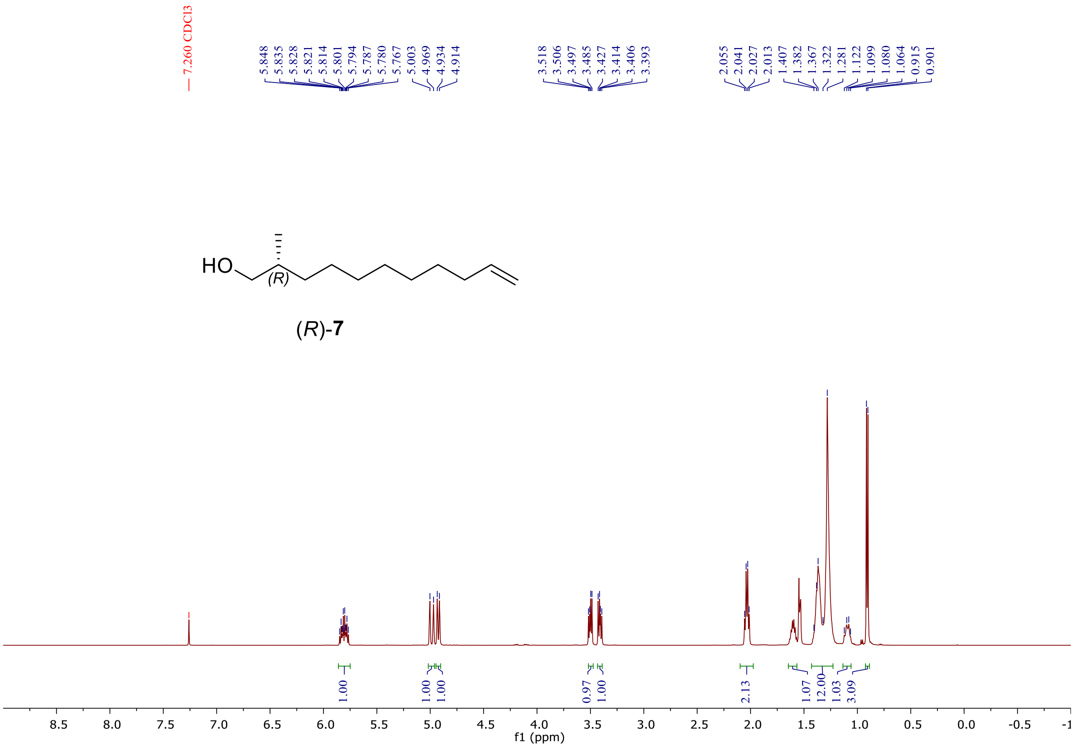

Figure S6. ^{13}C NMR Spectrum of compound (*R*)-**7** (125 MHz, $CDCl_3$)

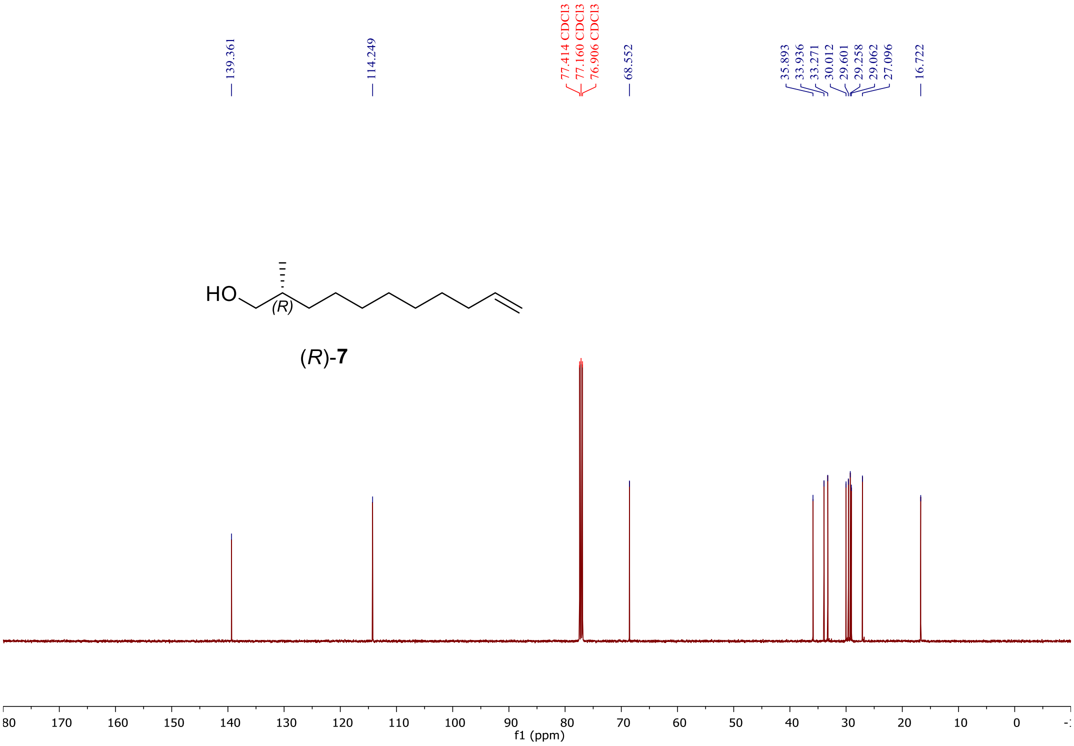

Figure S7. ^{1}H NMR Spectrum of compound (*S*)-**5** (500 MHz, $CDCl_3$)

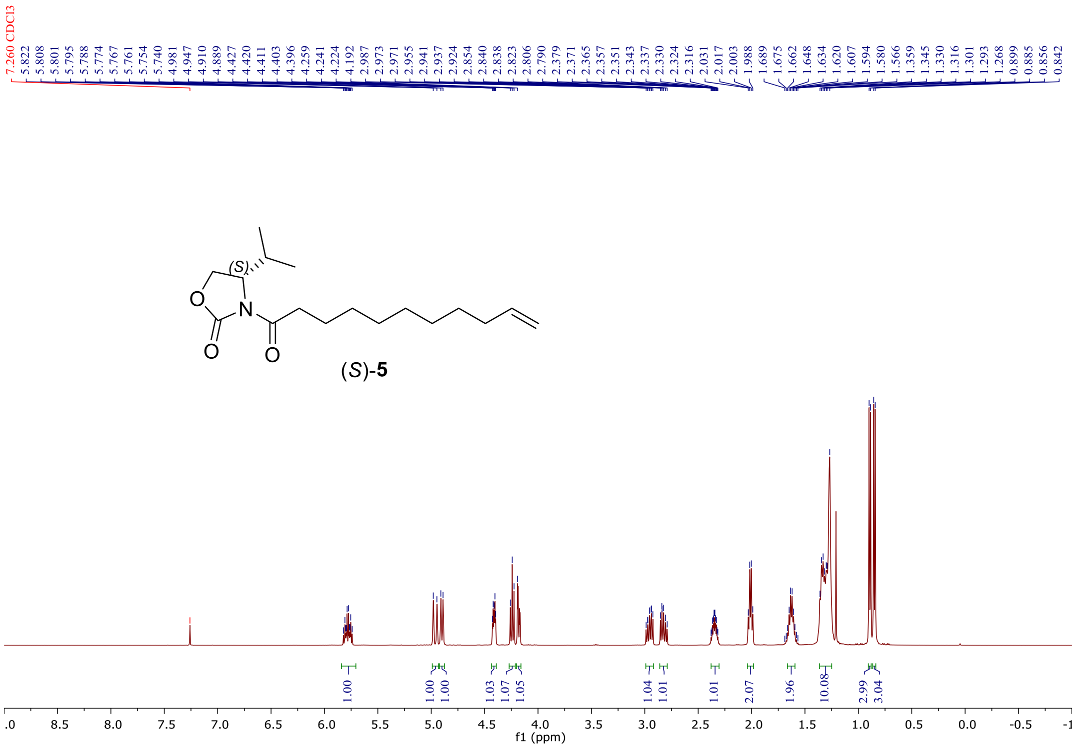

Figure S8. ^{13}C NMR Spectrum of compound (*S*)-**5** (125 MHz, $CDCl_3$)

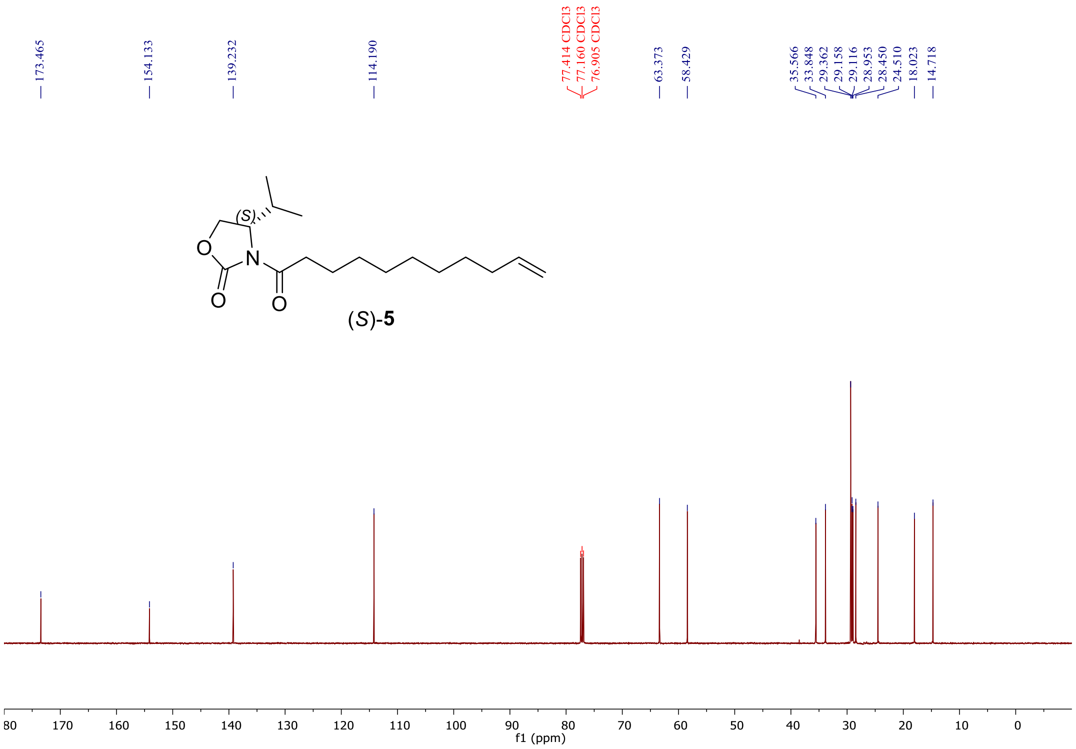

Figure S9. ^{1}H NMR Spectrum of compound (*S*, *S*)-**6** (500 MHz, $CDCl_3$)

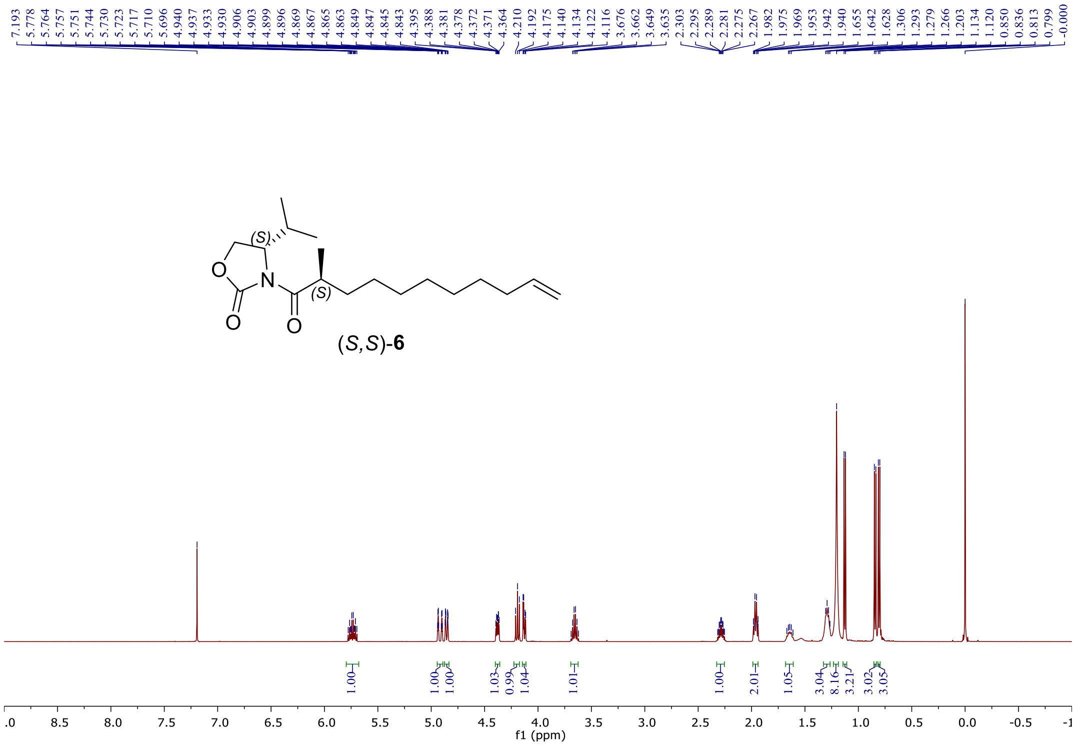

Figure S10. ^{13}C NMR Spectrum of compound (*S*, *S*)-**6** (125 MHz, $CDCl_3$)

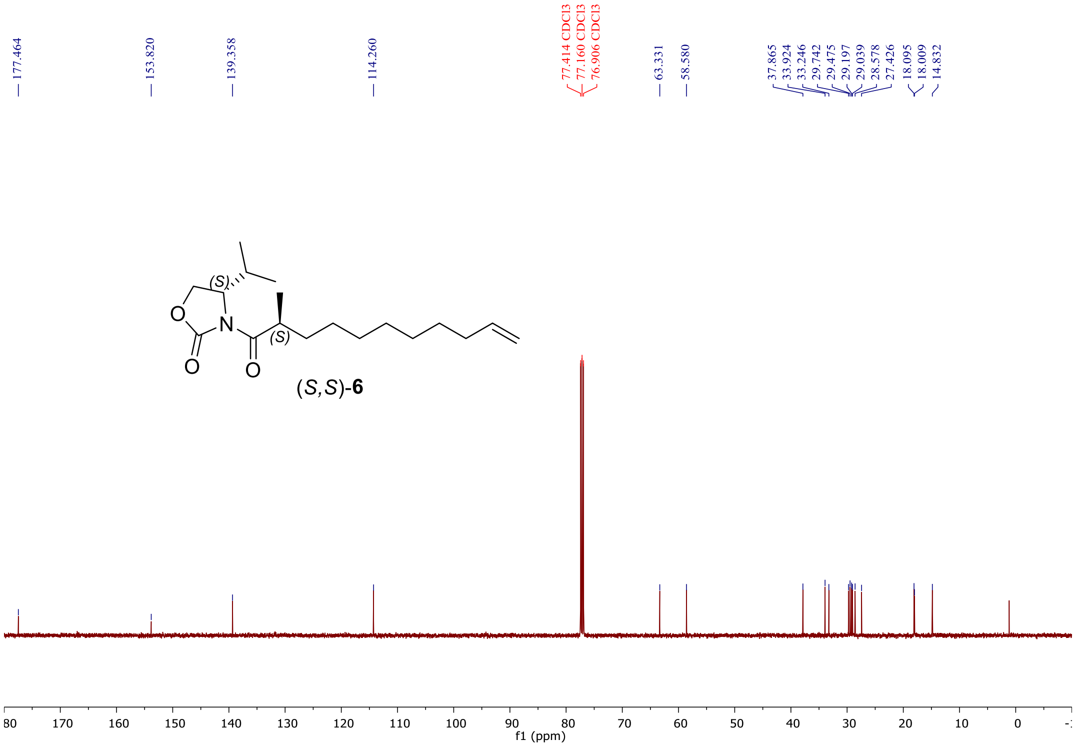

Figure S11. ^{1}H NMR Spectrum of compound (*S*)-**7** (500 MHz, $CDCl_3$)

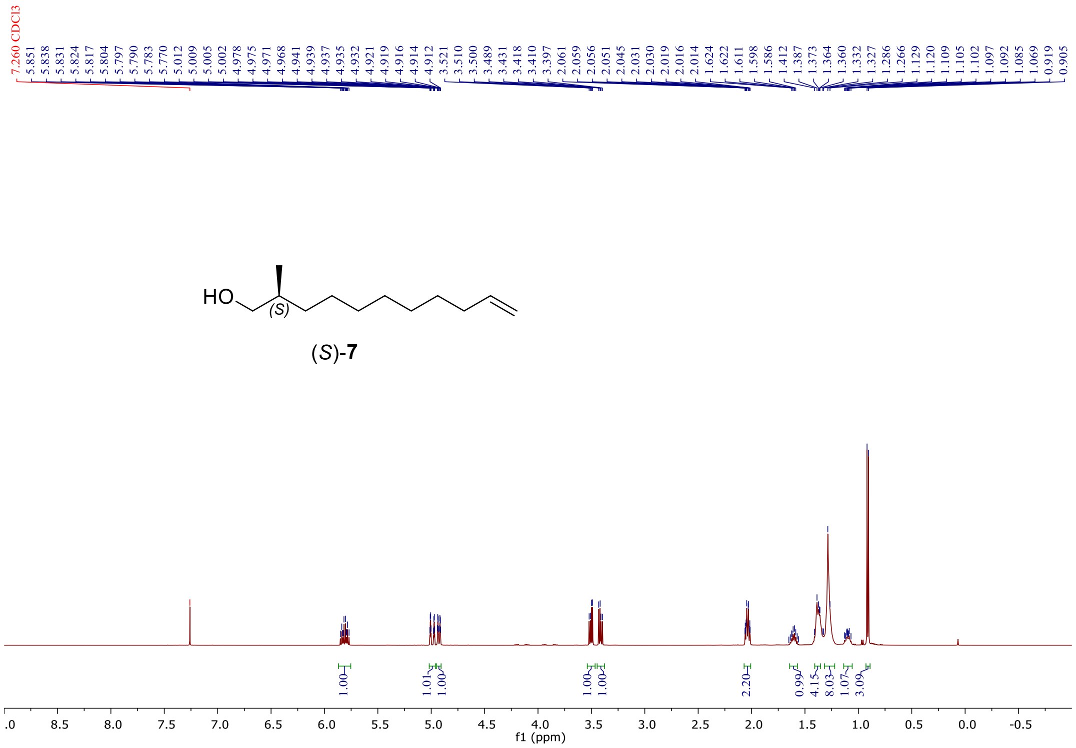

Figure S12. ^{13}C NMR Spectrum of compound (*S*)-**7** (125 MHz, $CDCl_3$)

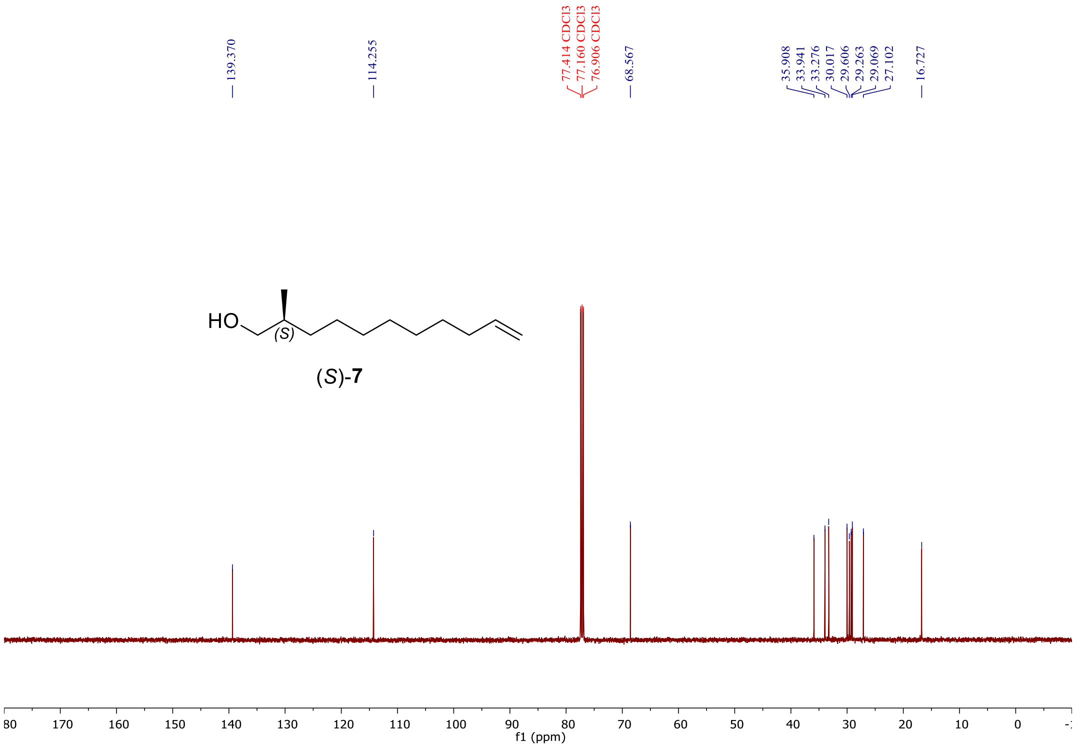

Figure S13. ^{1}H NMR Spectrum of compound (*S*)-**9** (500 MHz, $CDCl_3$)

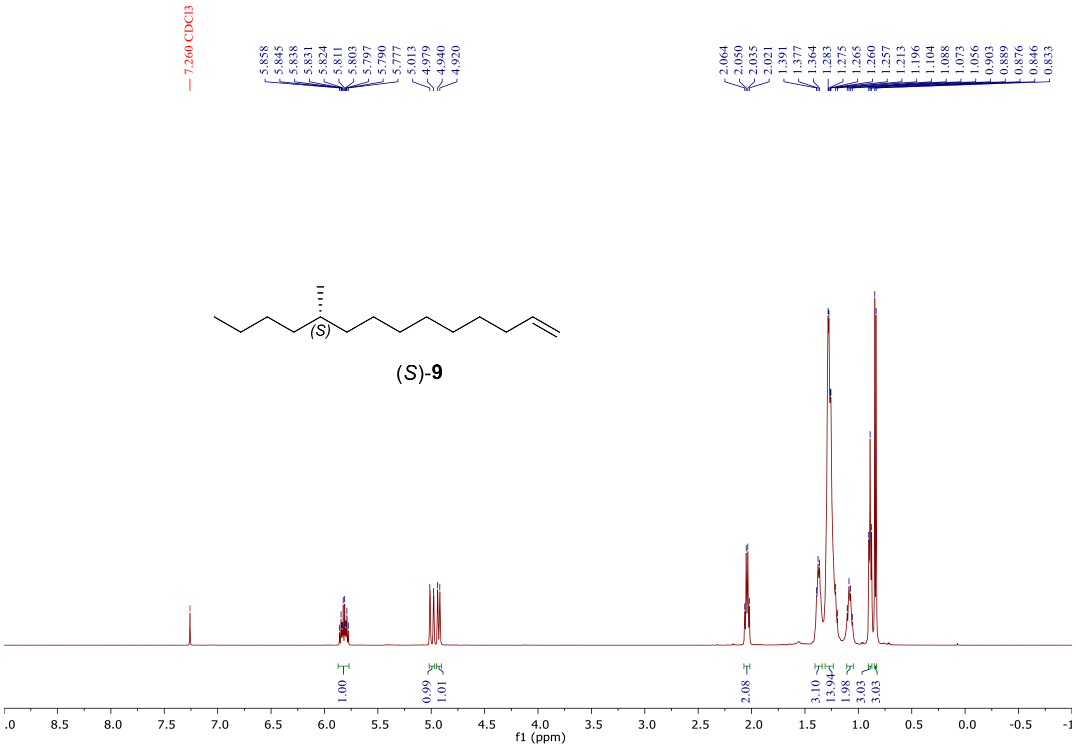

Figure S14. ^{13}C NMR Spectrum of compound (*S*)-**9** (125 MHz, $CDCl_3$)

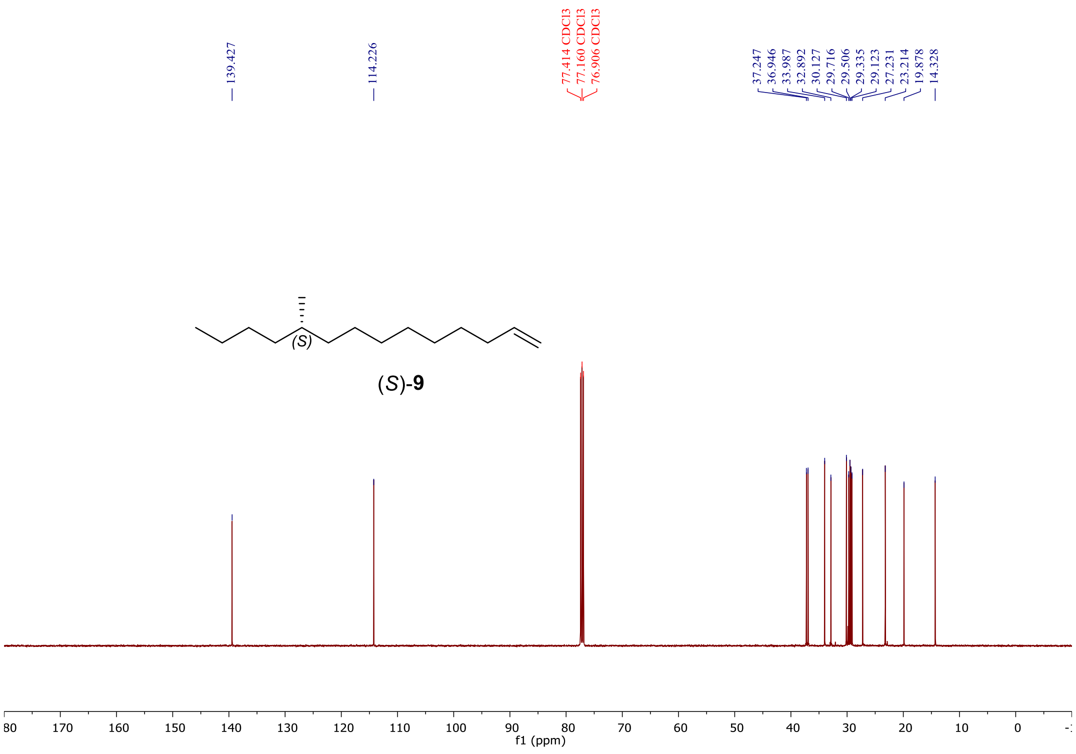

Figure S15. ^{1}H NMR Spectrum of compound (*S*)-**10** (500 MHz, $CDCl_3$)

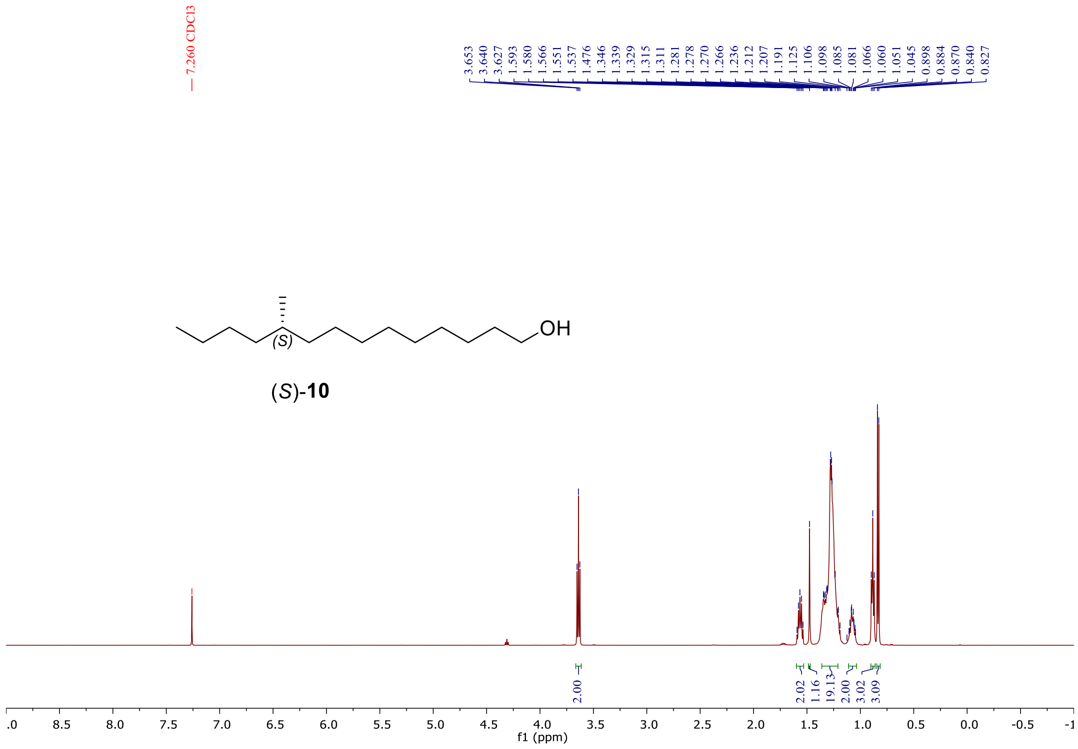

Figure S16. ^{13}C NMR Spectrum of compound (*S*)-**10** (125 MHz, $CDCl_3$)

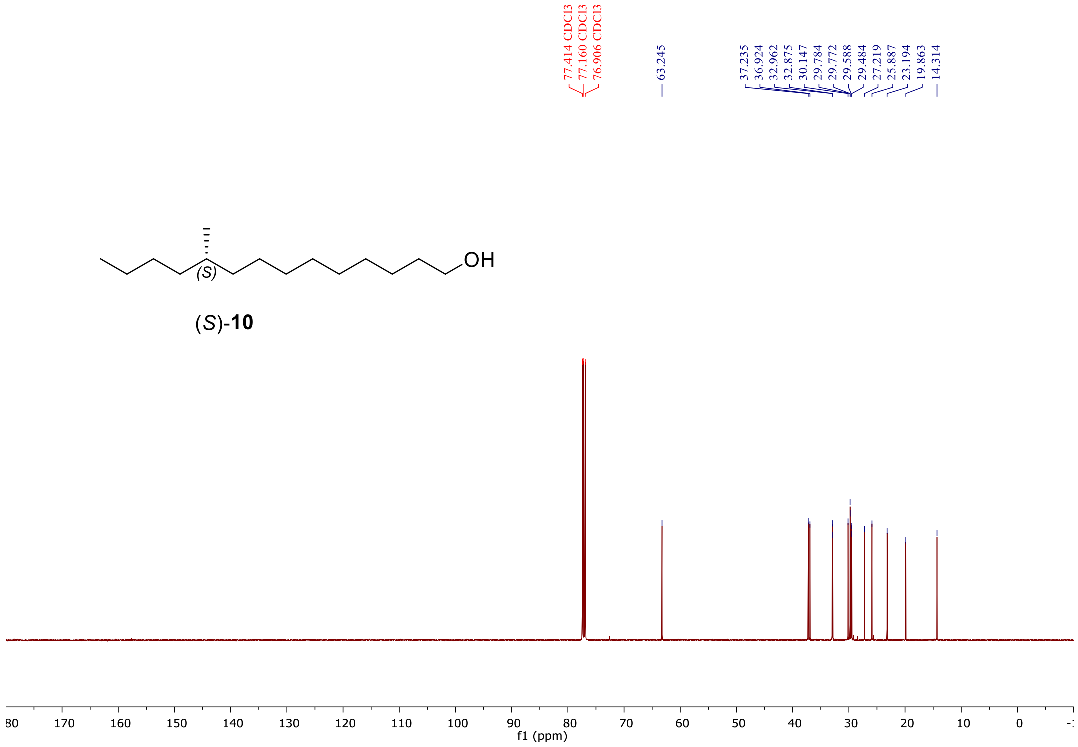

Figure S17. ^{1}H NMR Spectrum of compound (*S*)-**12** (500 MHz, $CDCl_3$)

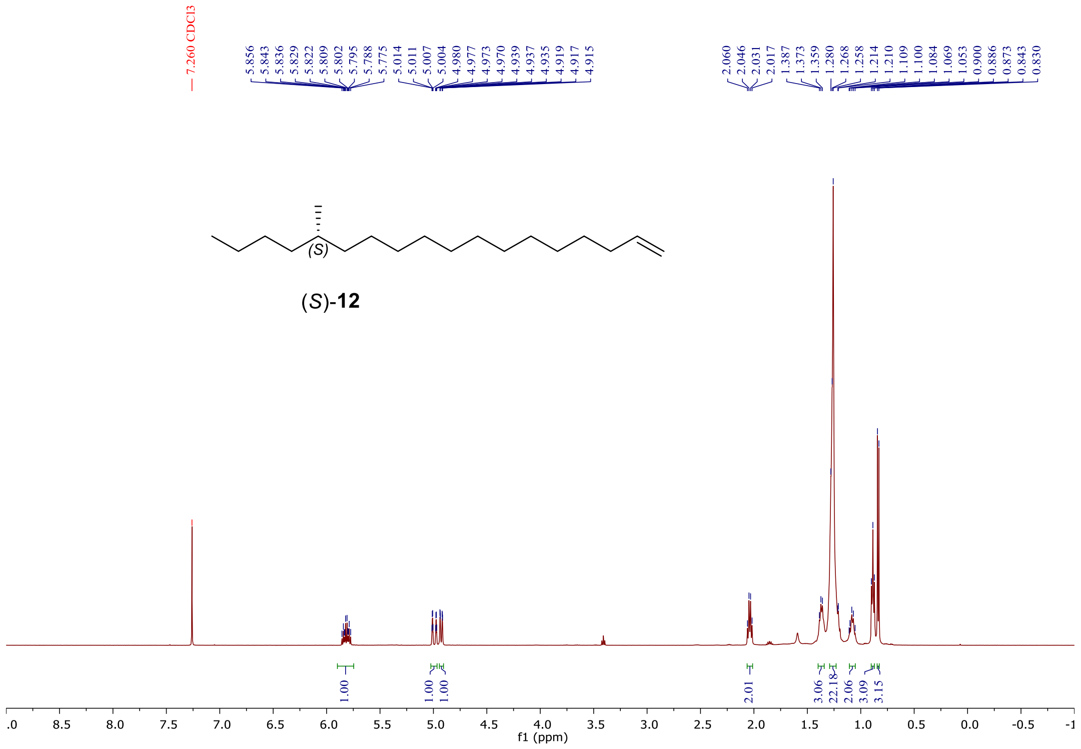

Figure S18. ^{13}C NMR Spectrum of compound (*S*)-**12** (125 MHz, $CDCl_3$)

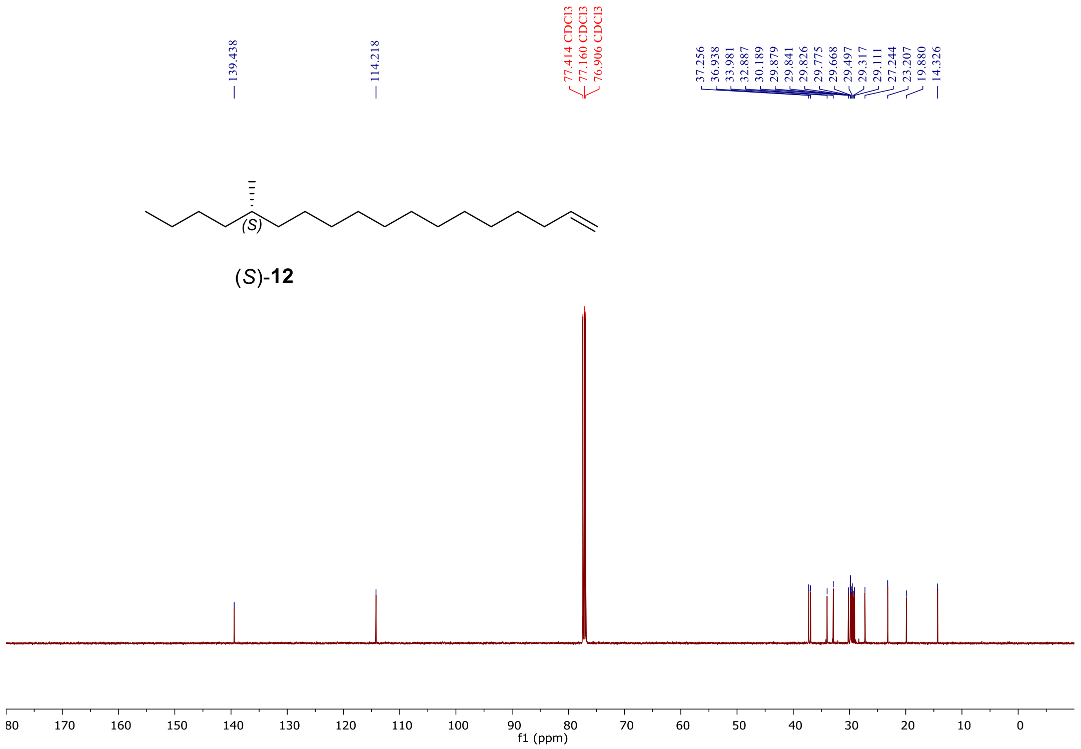

Figure S19. ^{1}H NMR Spectrum of compound (*R*)-**9** (500 MHz, $CDCl_3$)

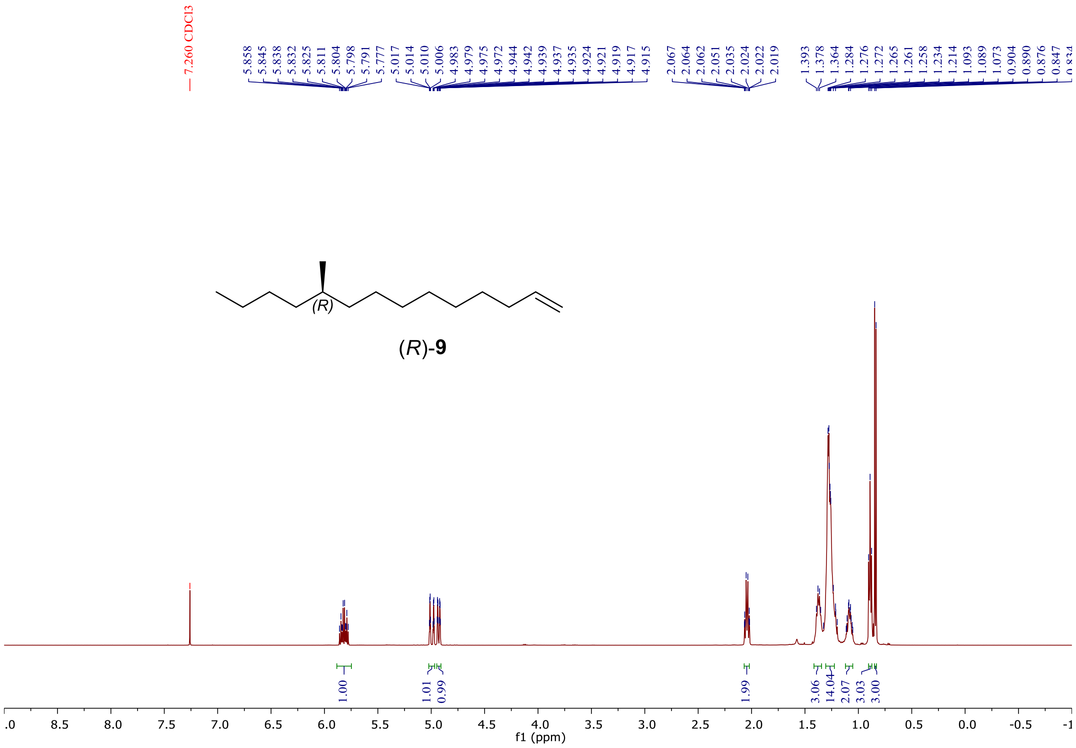

Figure S20. ^{13}C NMR Spectrum of compound (*R*)-**9** (125 MHz, $CDCl_3$)

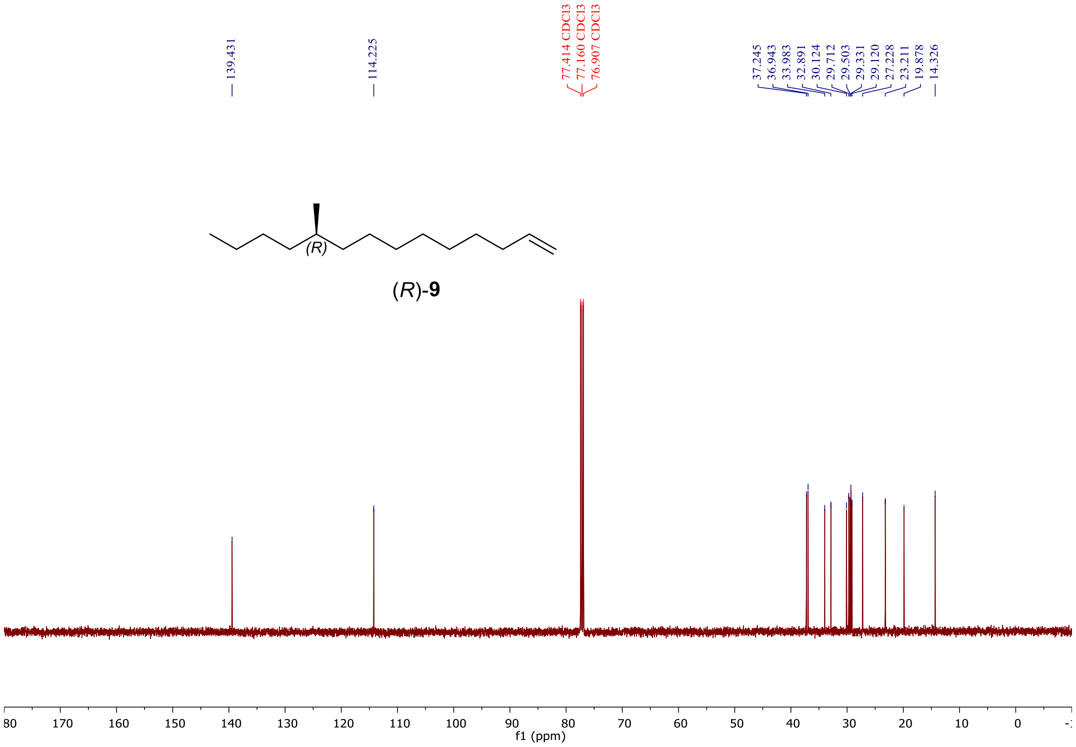

Figure S21. ^{1}H NMR Spectrum of compound (*R*)-**10** (500 MHz, $CDCl_3$)

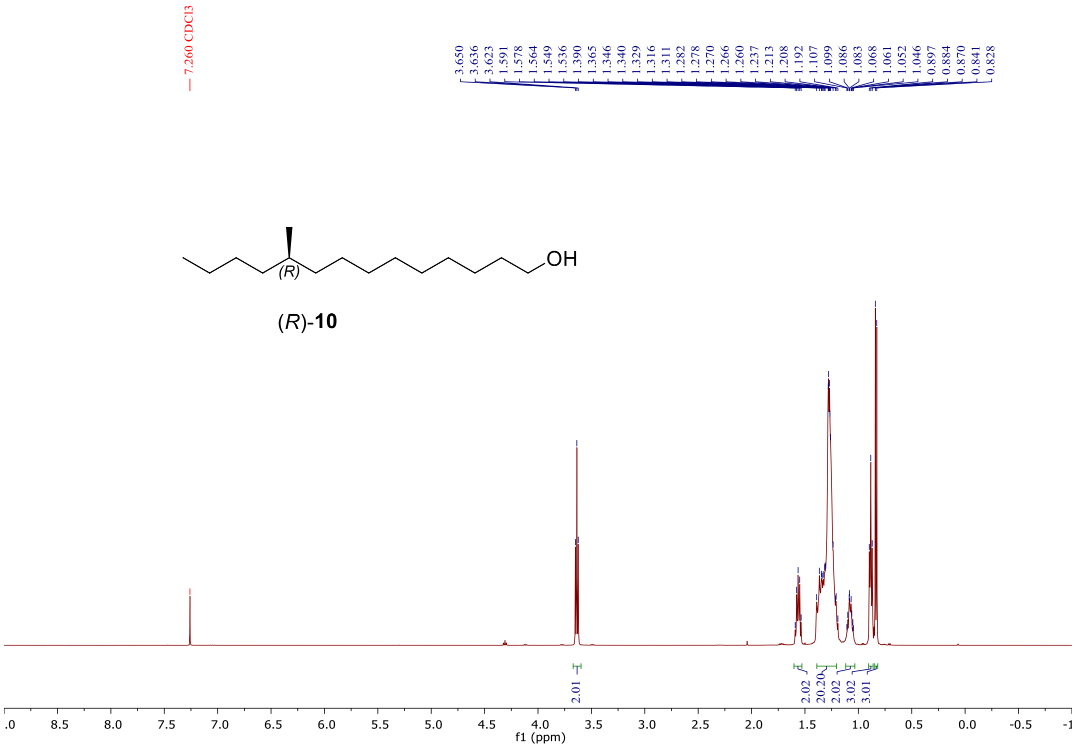

Figure S22. ^{13}C NMR Spectrum of compound (*R*)-**10** (125 MHz, $CDCl_3$)

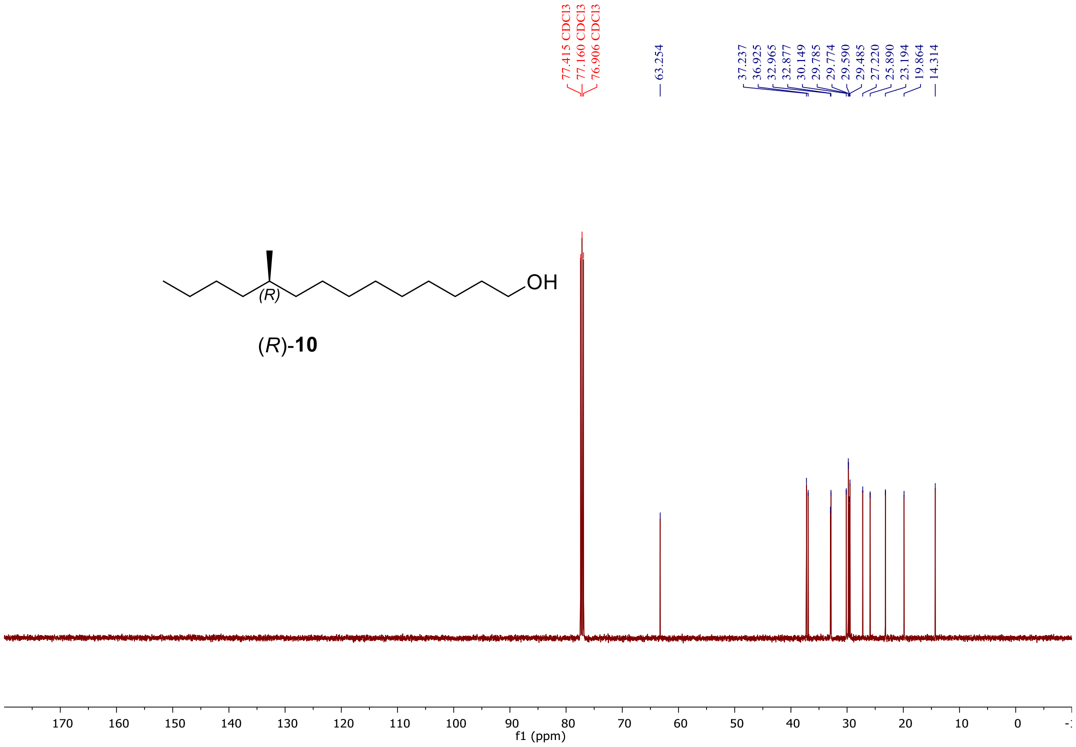

Figure S23. ^{1}H NMR Spectrum of compound (*R*)-**12** (500 MHz, $CDCl_3$)

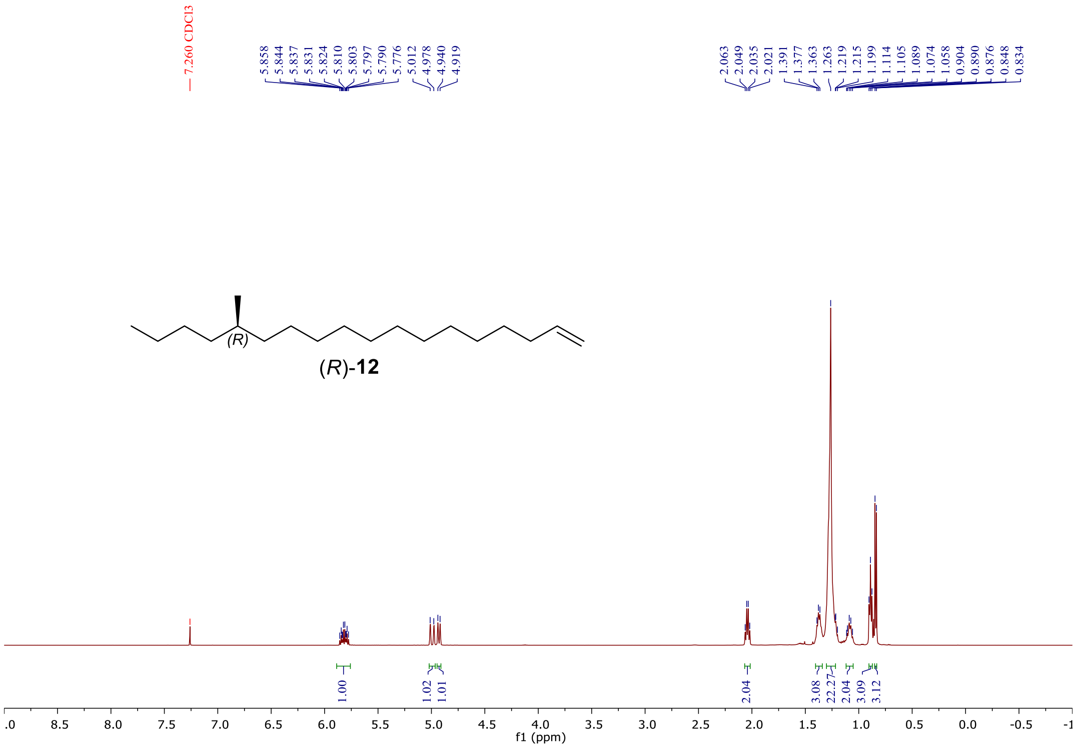

Figure S24. ^{13}C NMR Spectrum of compound (*R*)-**12** (125 MHz, $CDCl_3$)

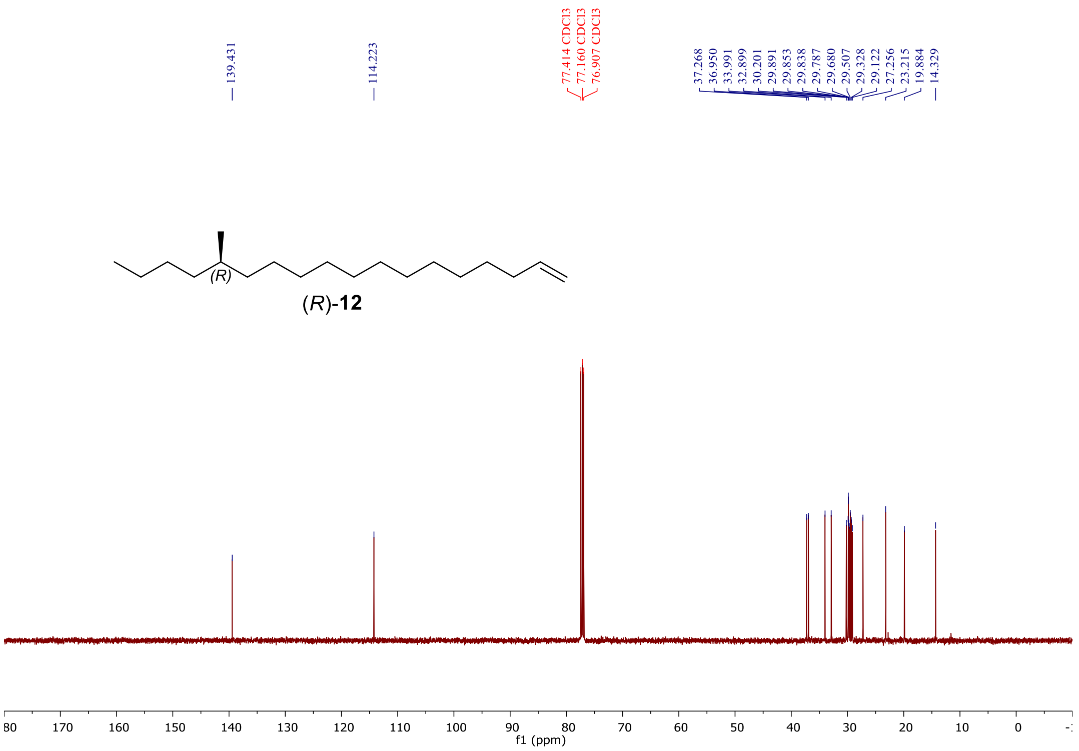

Figure S25. ¹H NMR Spectrum of compound (*S*)-**1** (500 MHz, $CDCl_3$)

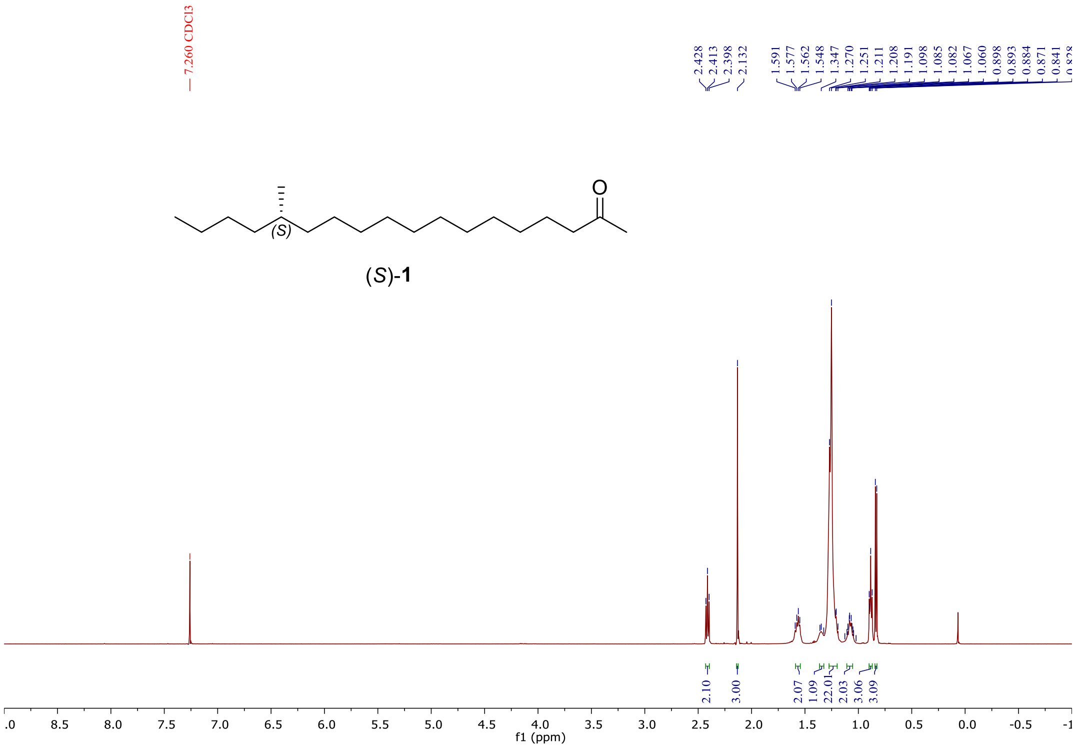

Figure S26. ¹³C NMR Spectrum of compound (*S*)-**1** (125 MHz, $CDCl_3$)

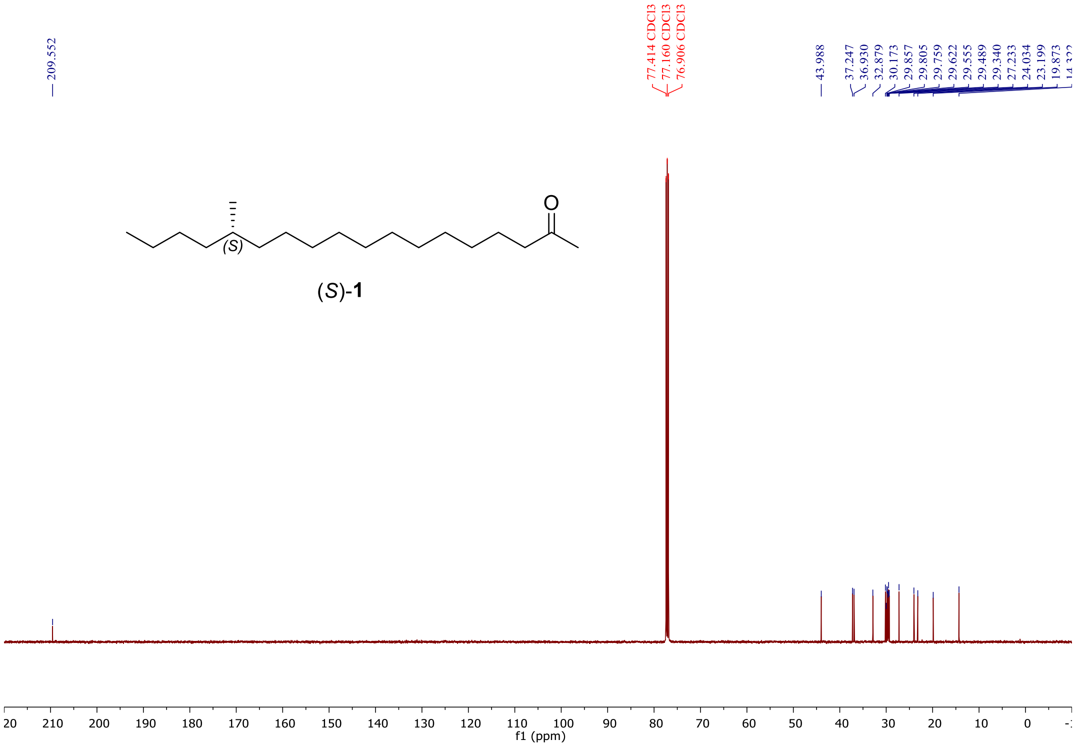

Figure S27. ^{1}H NMR Spectrum of compound (*R*)-**1** (500 MHz, $CDCl_3$)

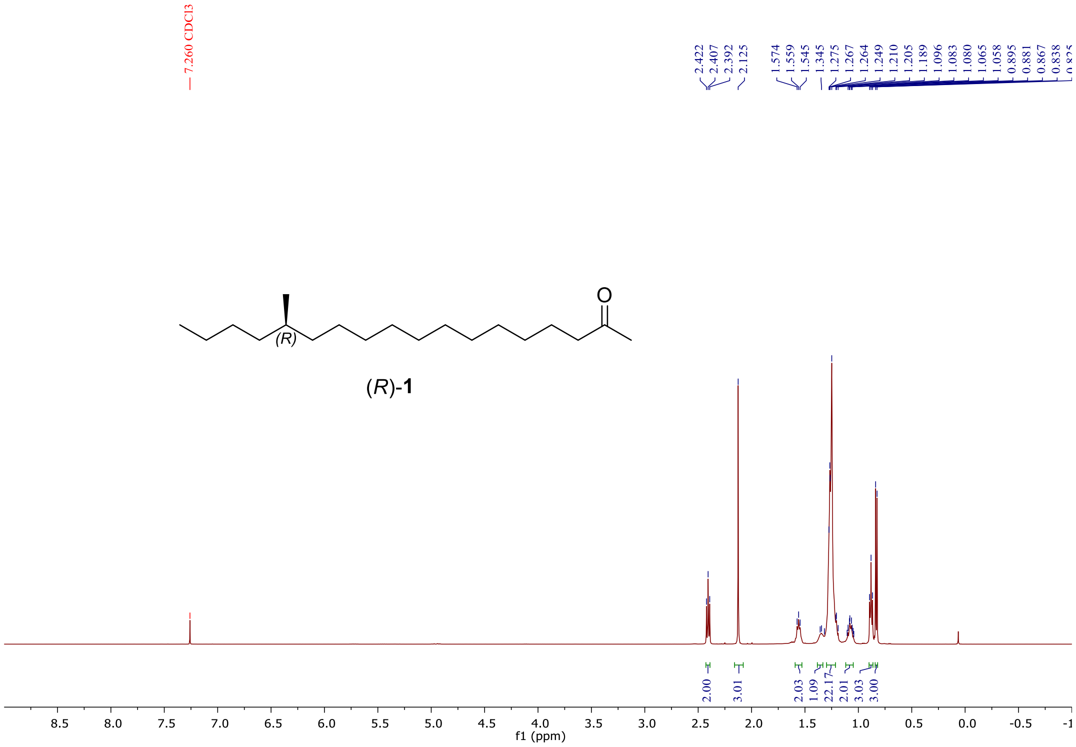

Figure S28. ^{13}C NMR Spectrum of compound (*R*)-**1** (125 MHz, $CDCl_3$)

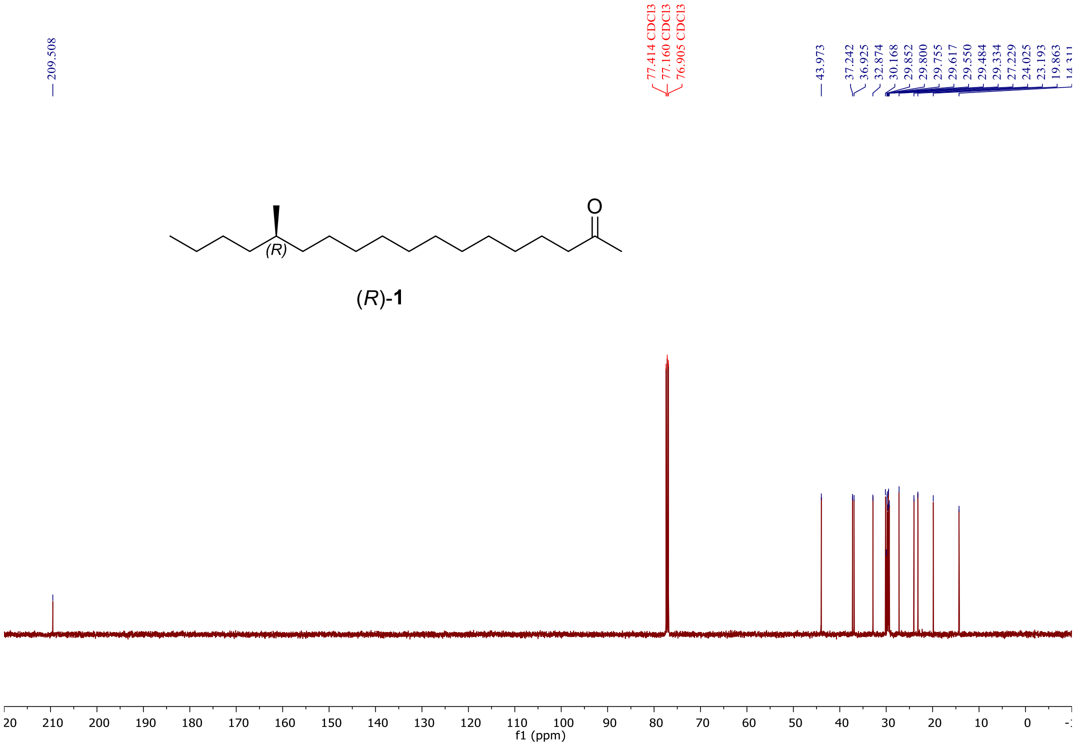

Figure S29. ^{1}H NMR Spectrum of compound (*S*)-**14** (500 MHz, $CDCl_3$)

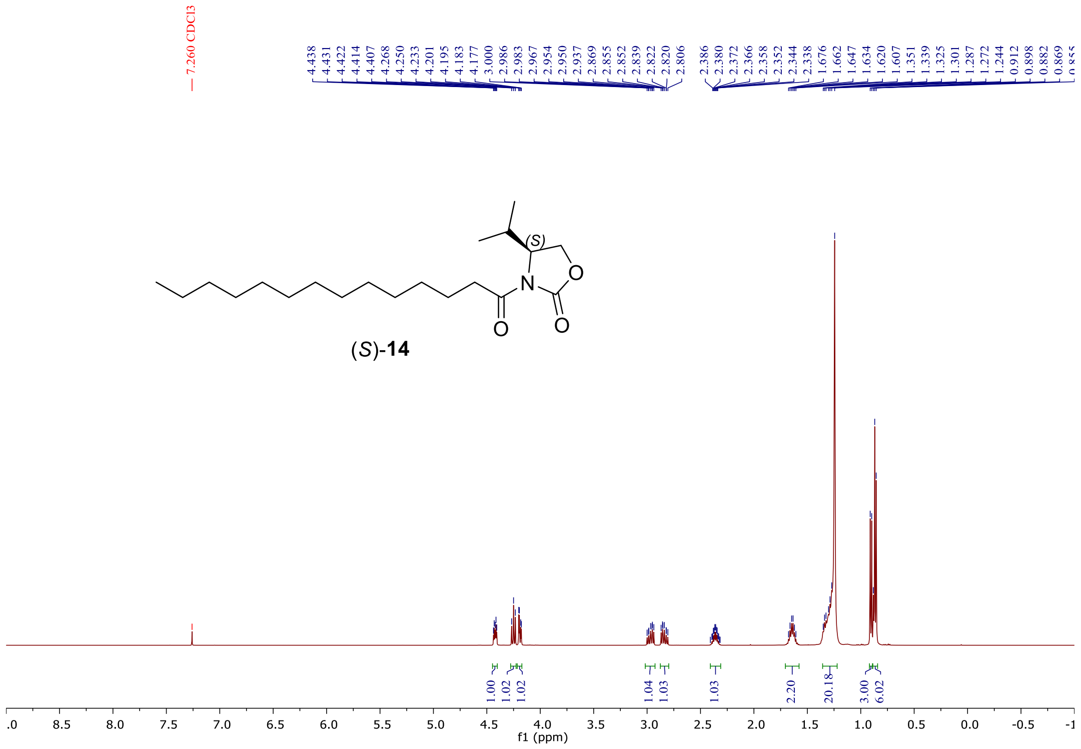

Figure S30. ^{13}C NMR Spectrum of compound (*S*)-**14** (125 MHz, $CDCl_3$)

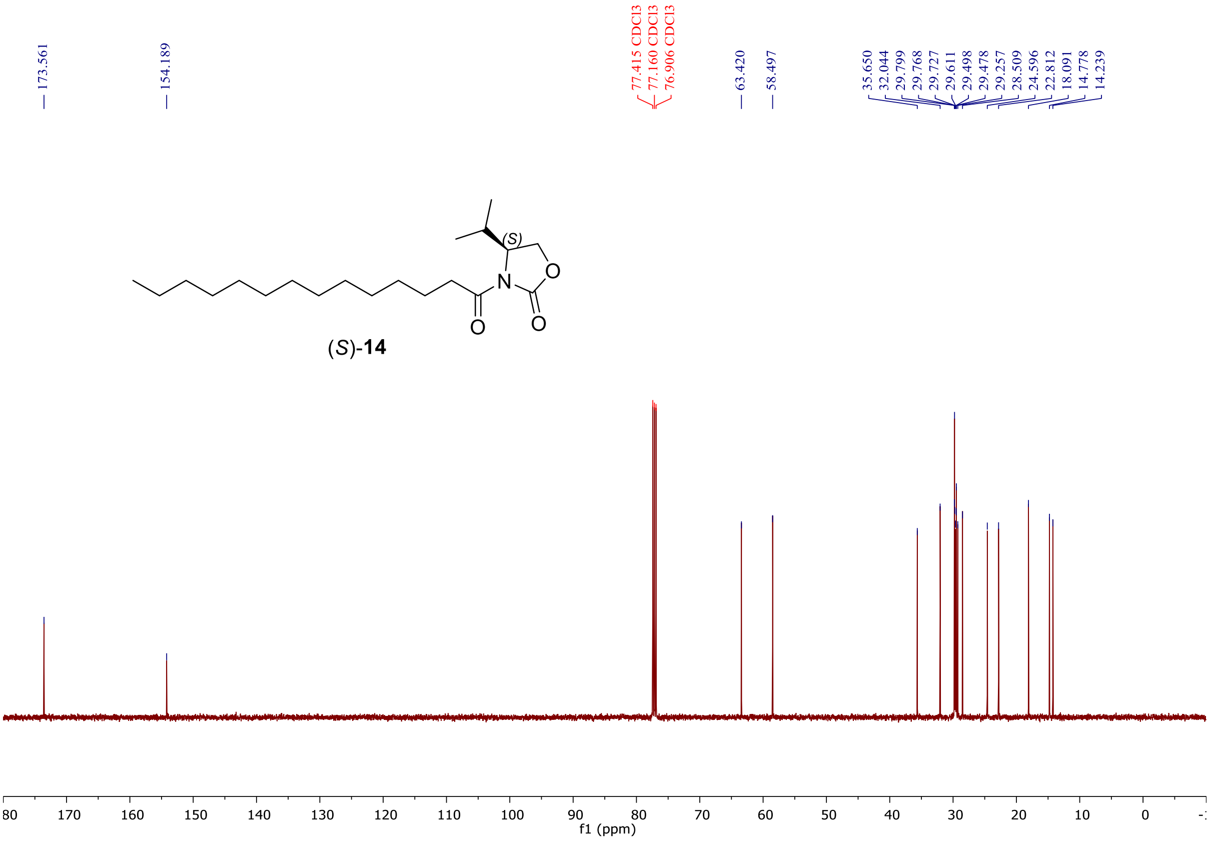

Figure S31. ^{1}H NMR Spectrum of compound (*S*,*S*)-**15** (500 MHz, $CDCl_3$)

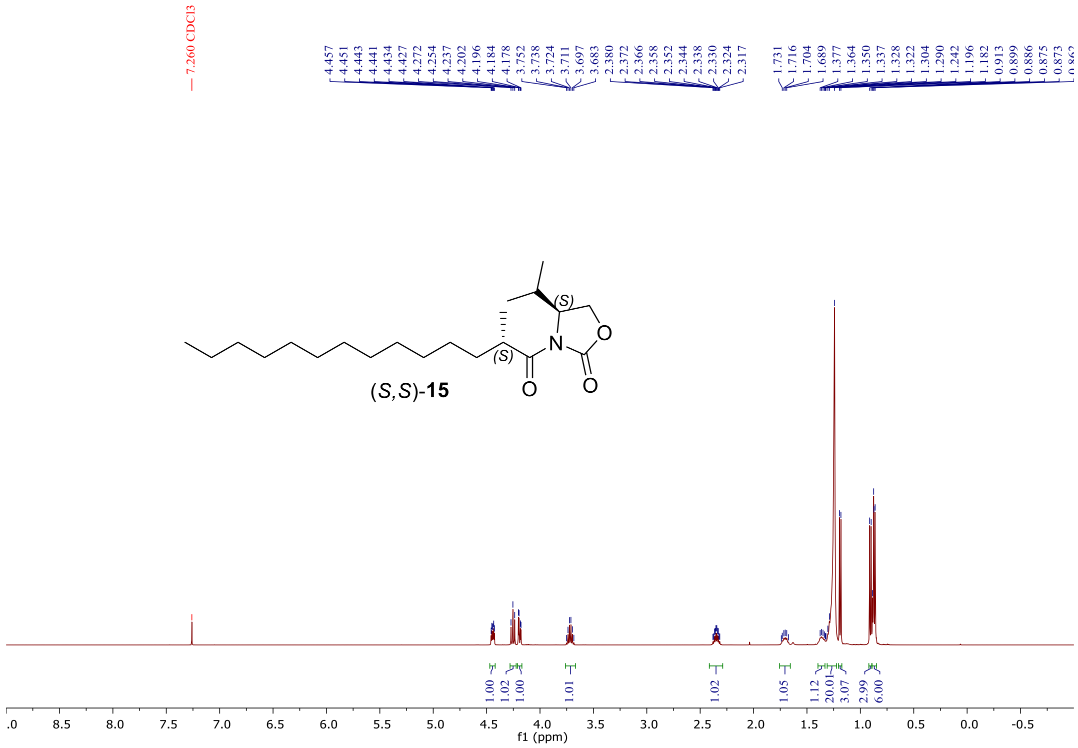

Figure S32. ^{13}C NMR Spectrum of compound (*S*,*S*)-**15** (125 MHz, $CDCl_3$)

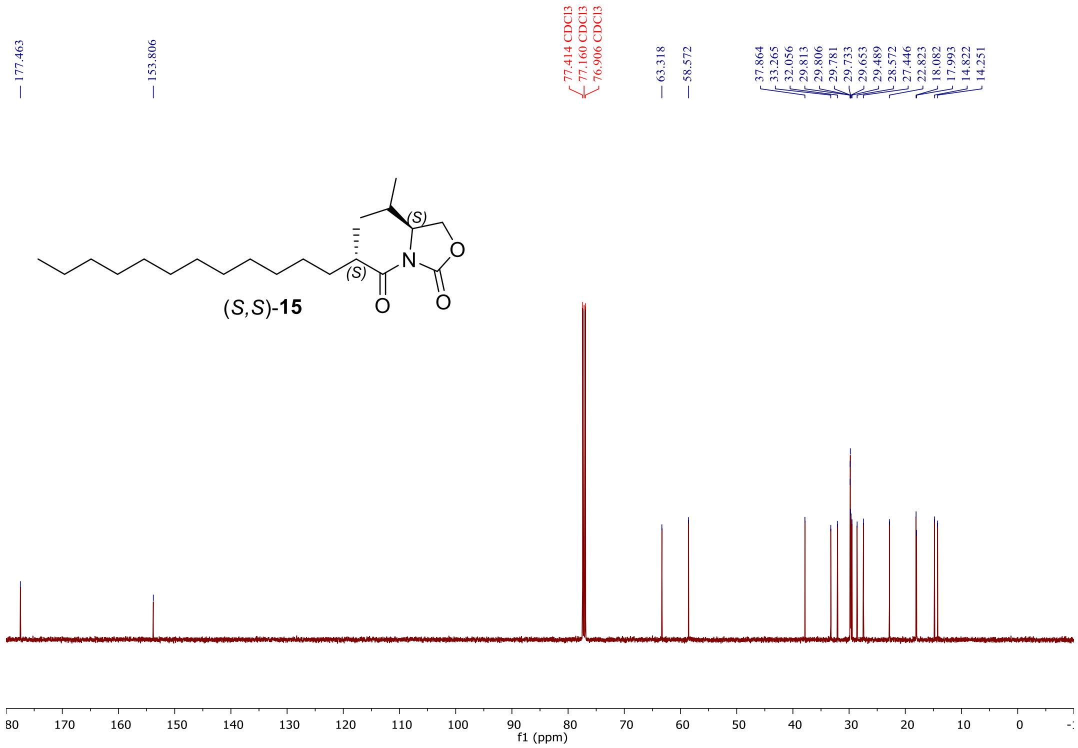

Figure S33. ^{1}H NMR Spectrum of compound (*S*)-**16** (500 MHz, $CDCl_3$)

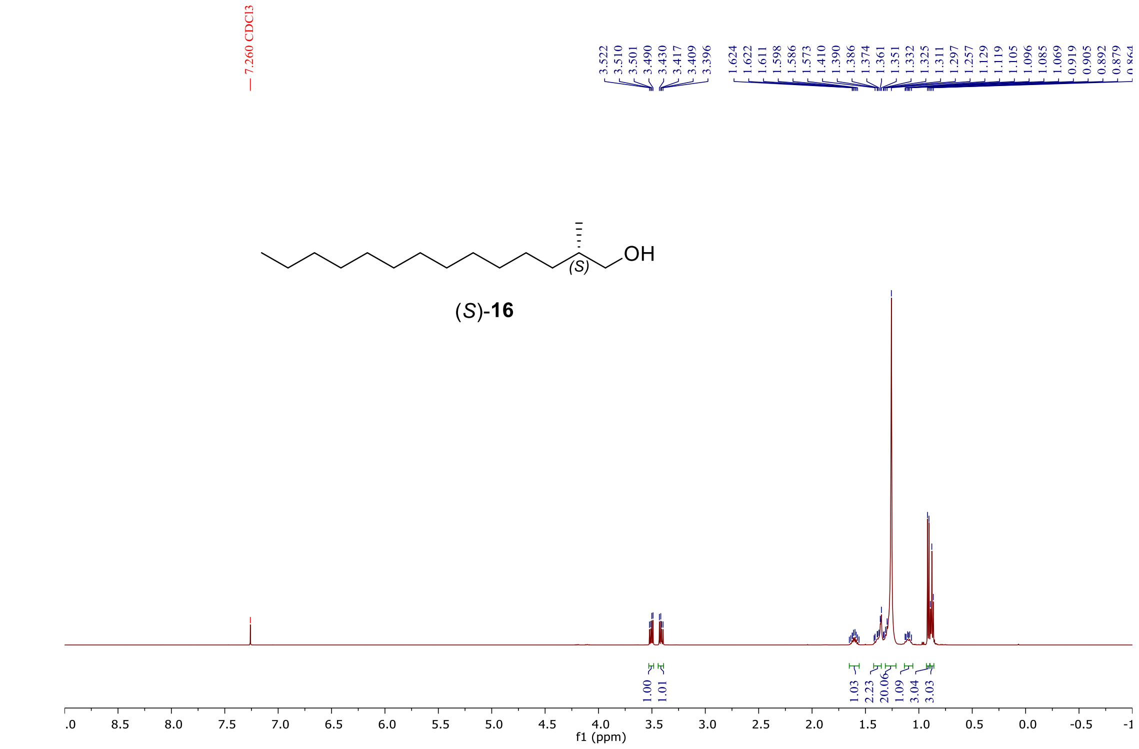

Figure S34. ^{13}C NMR Spectrum of compound (*S*)-**16** (125 MHz, $CDCl_3$)

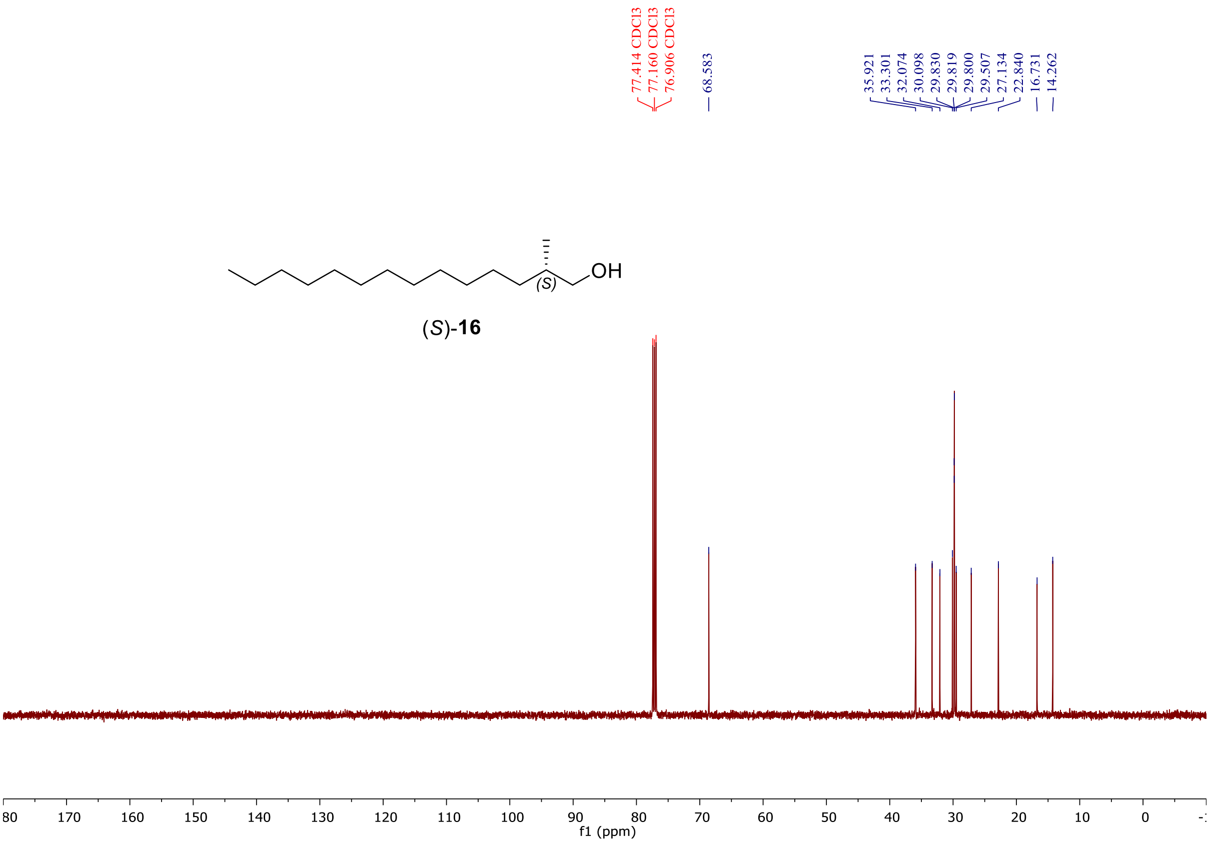

Figure S35. ^{1}H NMR Spectrum of compound (*R*)-**14** (500 MHz, $CDCl_3$)

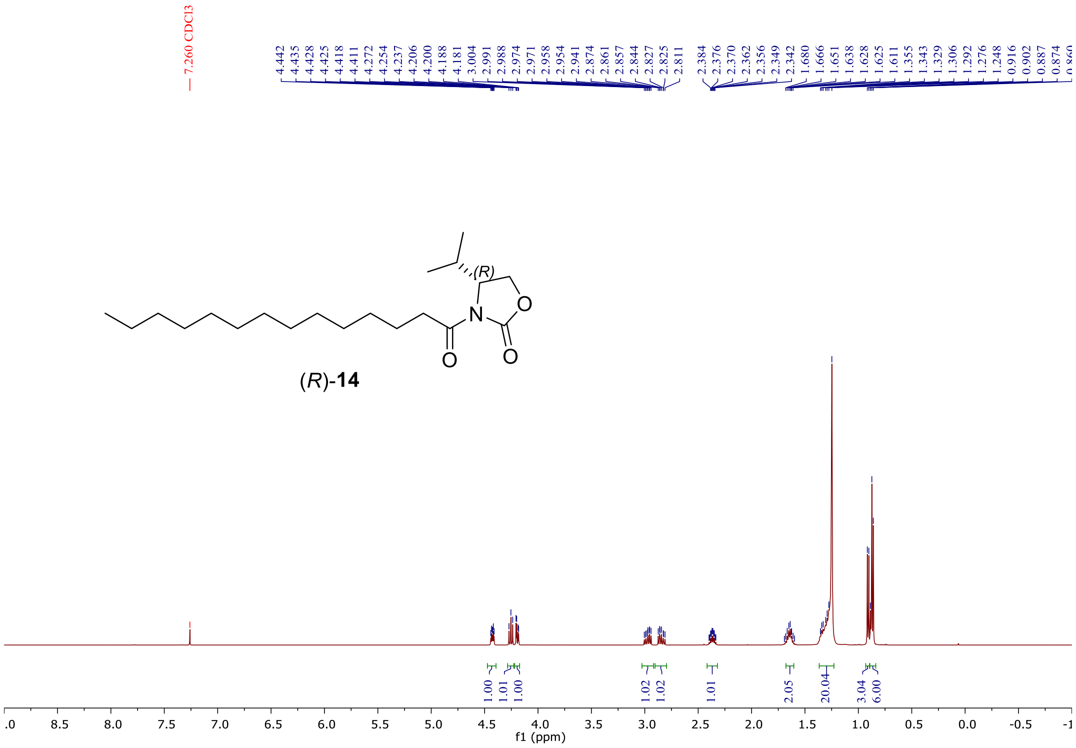

Figure S36. ^{13}C NMR Spectrum of compound (*R*)-**14** (125 MHz, $CDCl_3$)

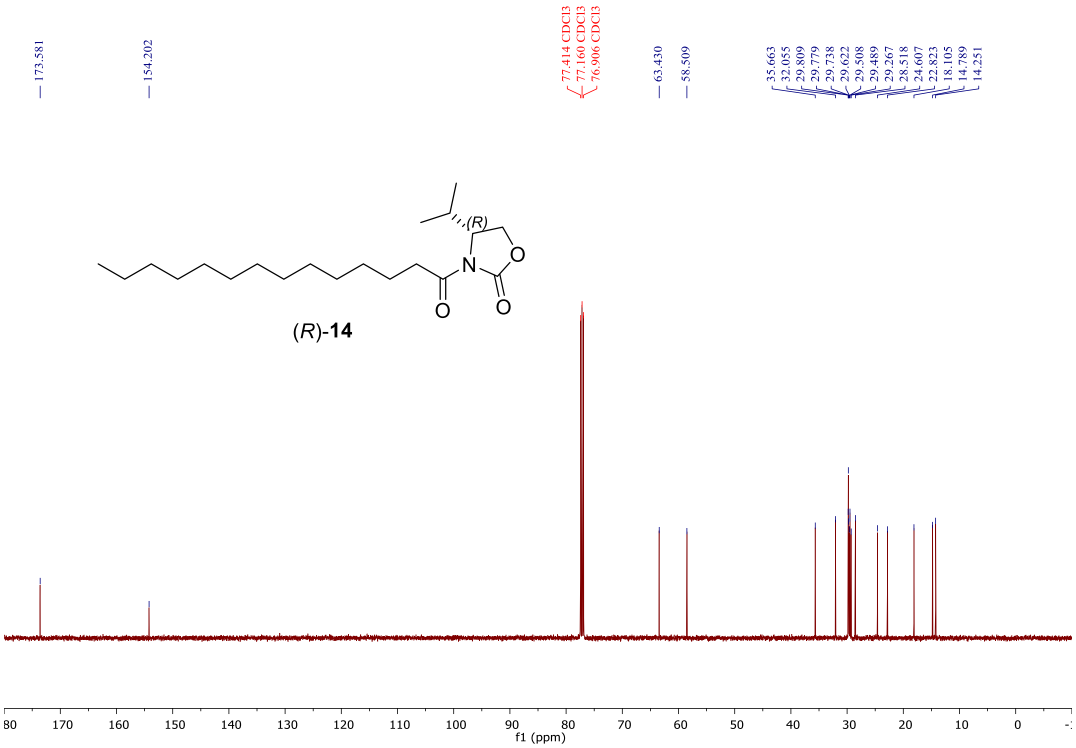

Figure S37. ^{1}H NMR Spectrum of compound (*R*,*R*)-**15** (500 MHz, $CDCl_3$)

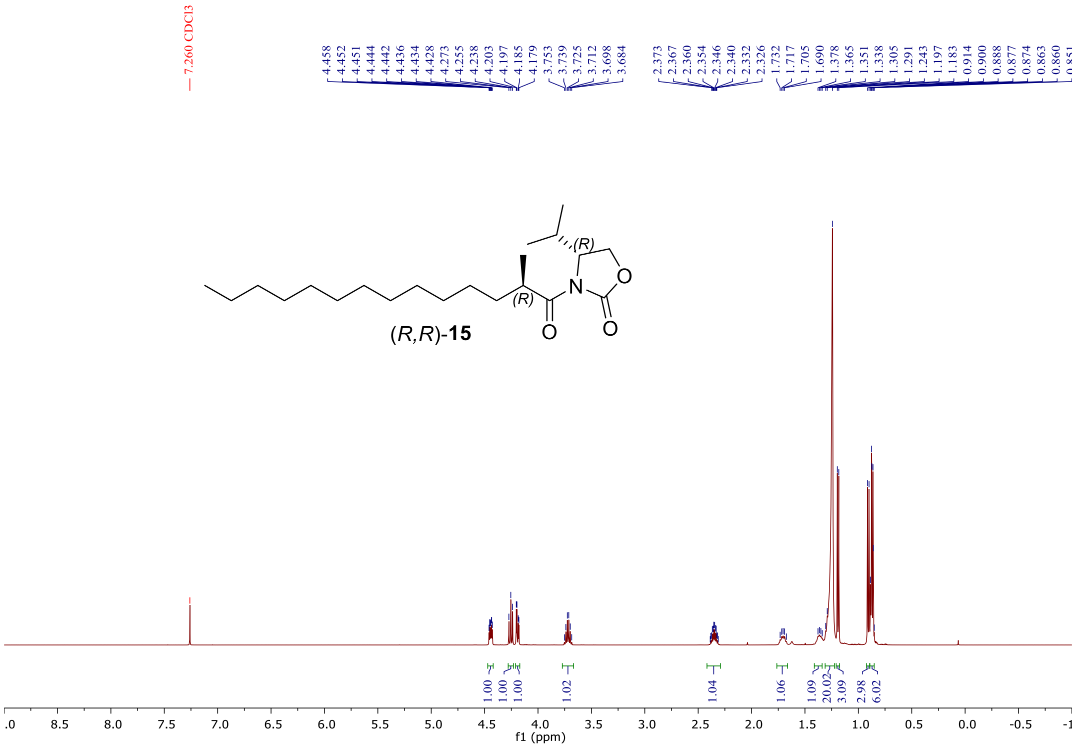

Figure S38. ^{13}C NMR Spectrum of compound (*R*,*R*)-**15** (125 MHz, $CDCl_3$)

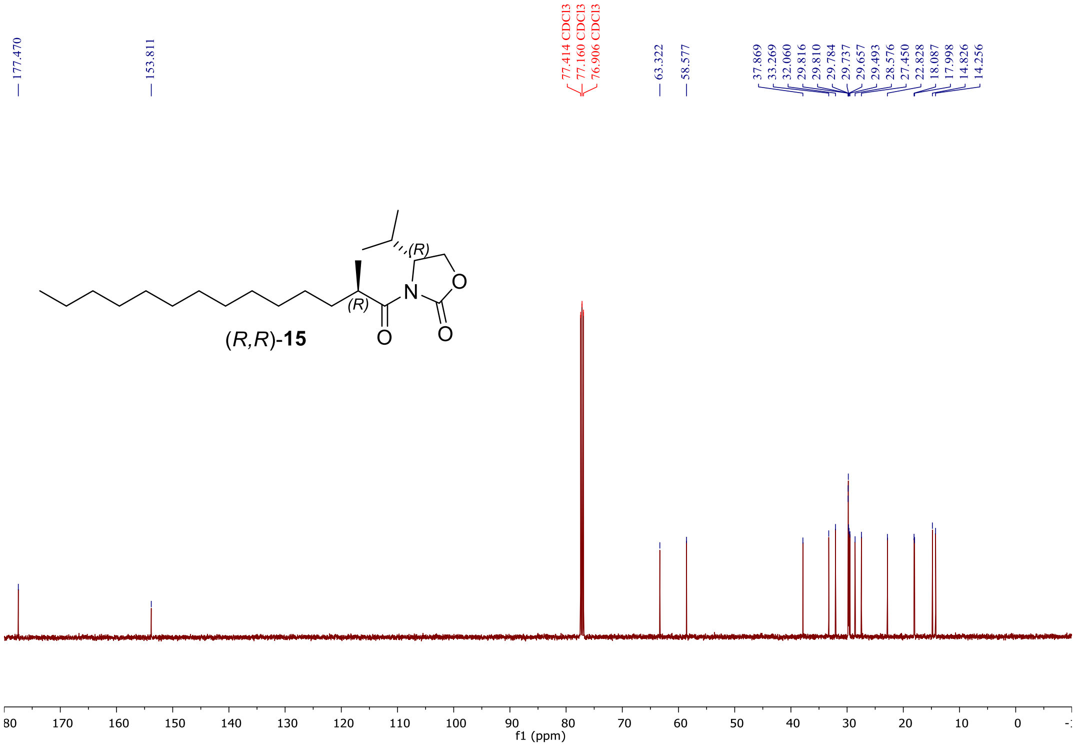

Figure S39. ^{1}H NMR Spectrum of compound (*R*)-**16** (500 MHz, $CDCl_3$)

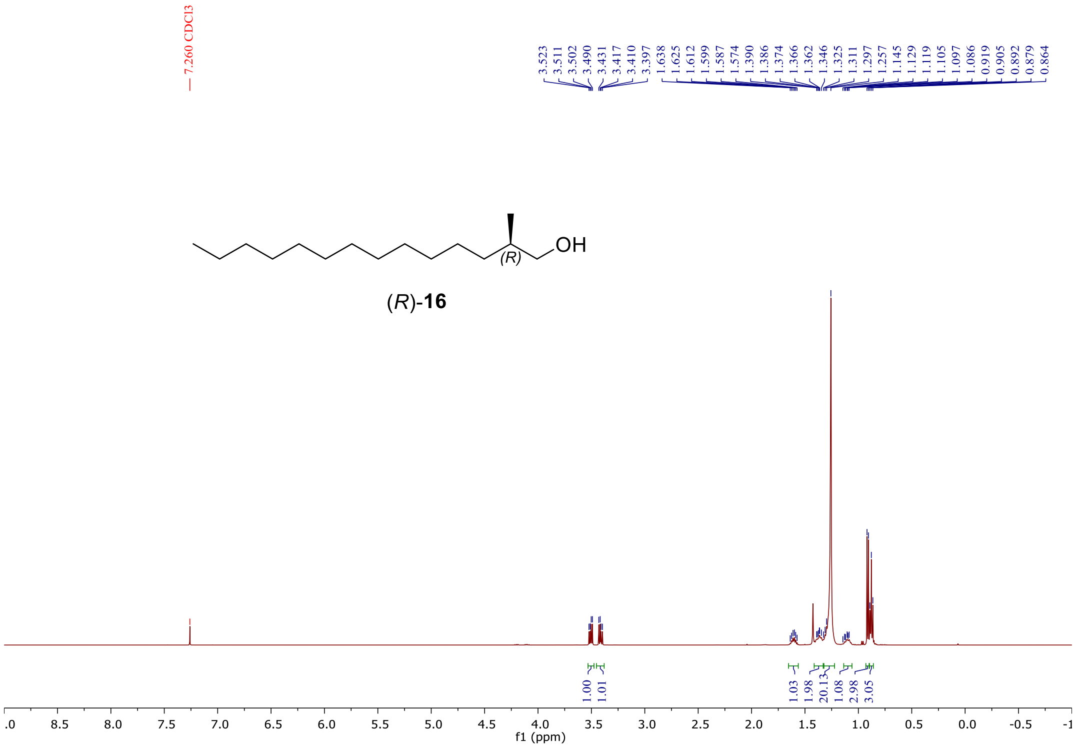

Figure S40. ^{13}C NMR Spectrum of compound (*R*)-**16** (125 MHz, $CDCl_3$)

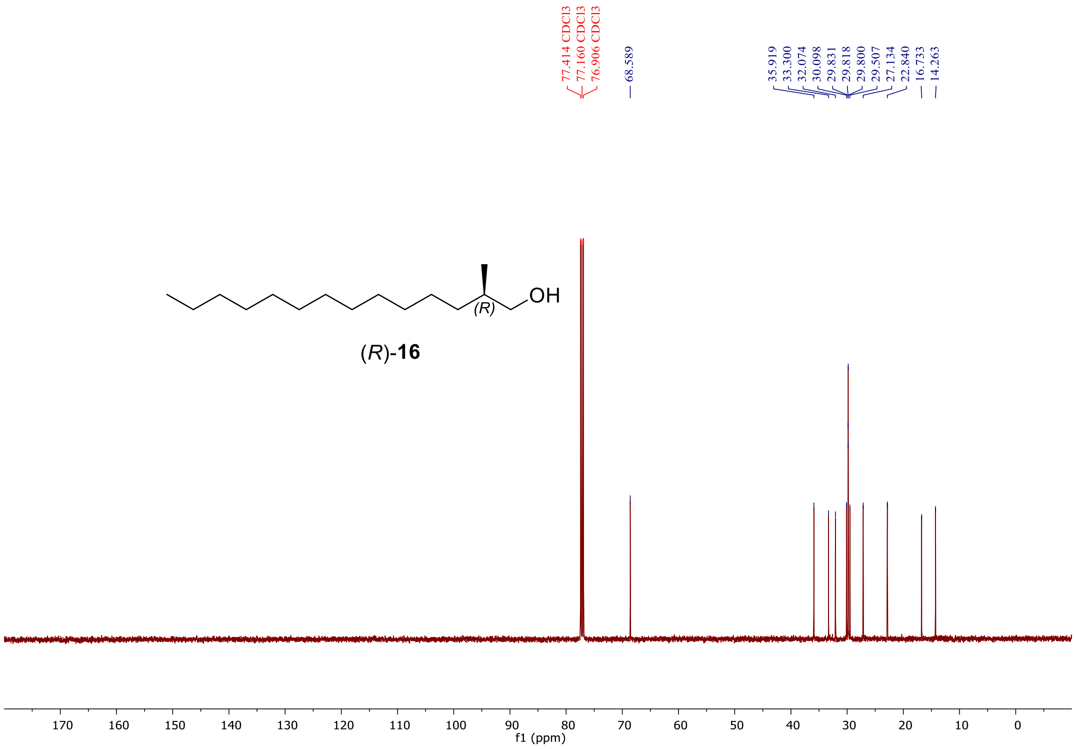

Figure S41. ^{1}H NMR Spectrum of compound (*S*)-**17** (500 MHz, $CDCl_3$)

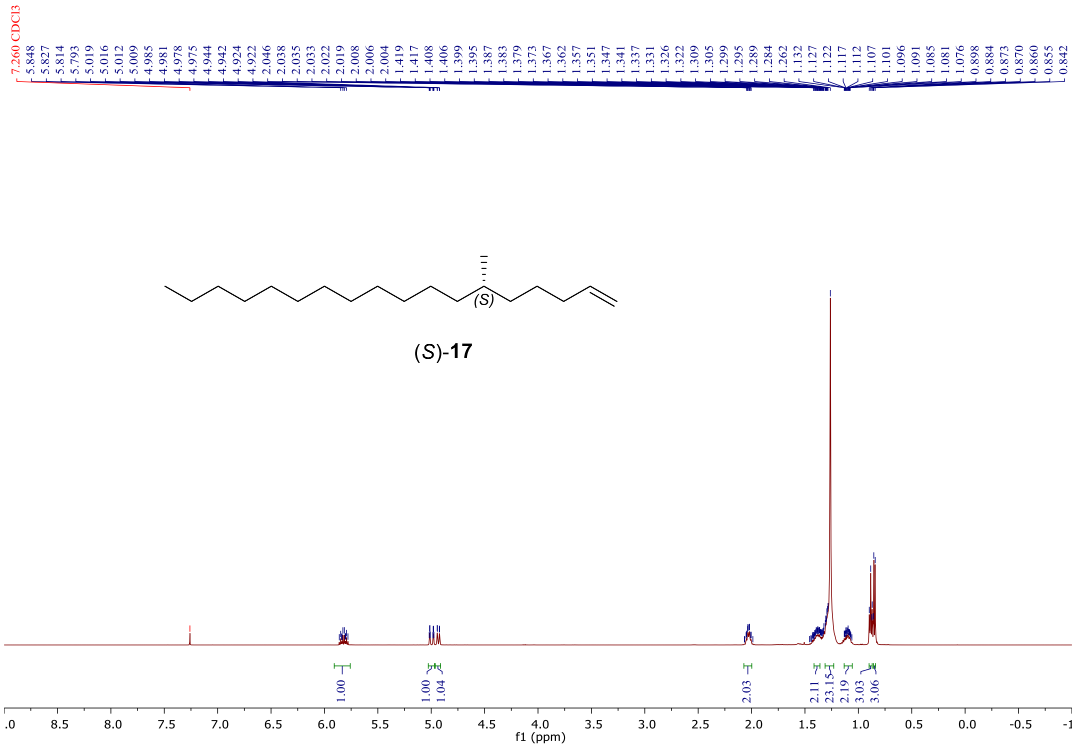

Figure S42. ^{13}C NMR Spectrum of compound (*S*)-**17** (125 MHz, $CDCl_3$)

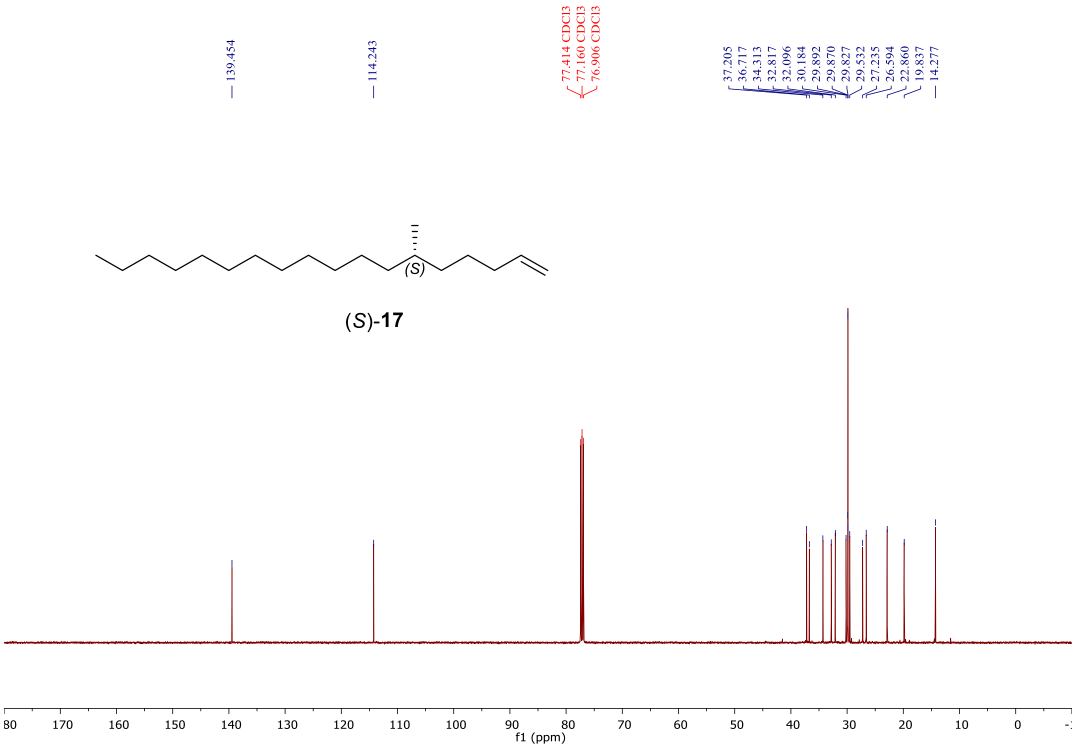

Figure S43. ^1H NMR Spectrum of compound (*R*)-**17** (500 MHz, $CDCl_3$)

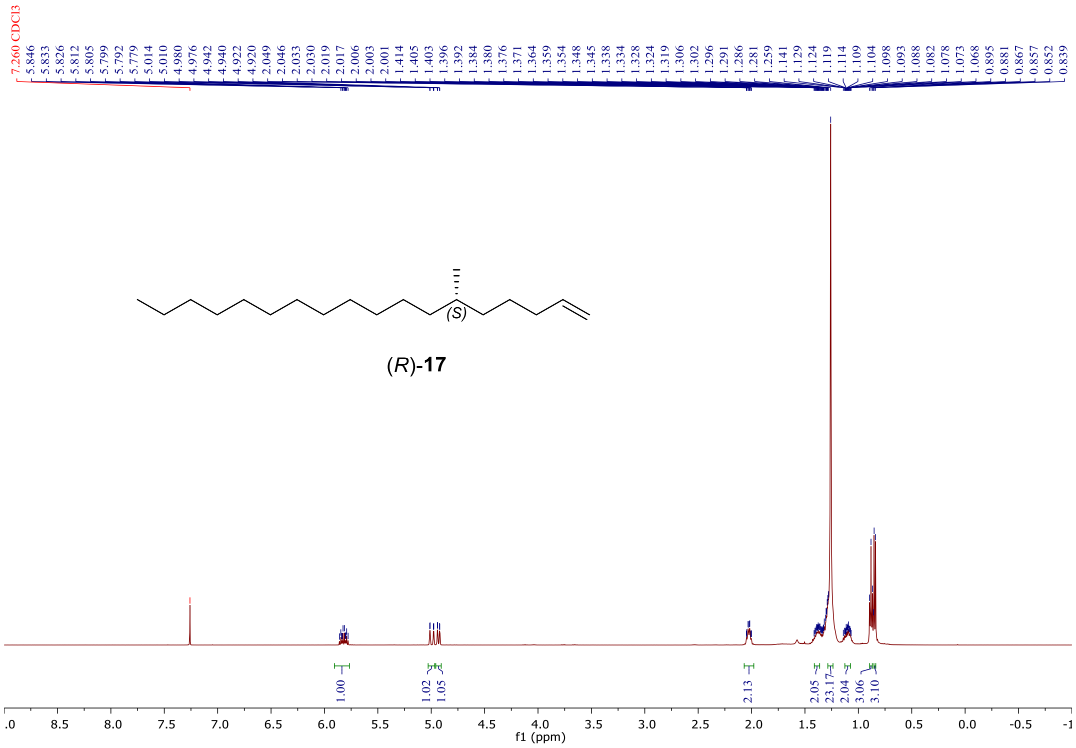

Figure S44. ^{13}C NMR Spectrum of compound (*R*)-**17** (125 MHz, $CDCl_3$)

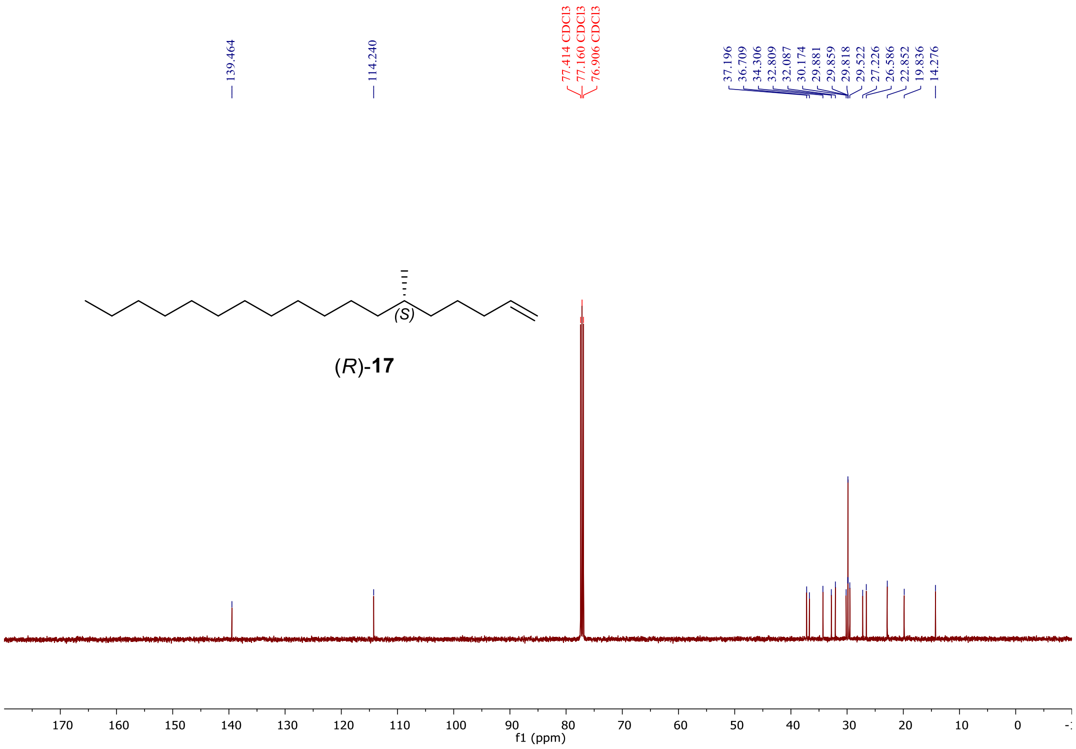

Figure S45. ^{1}H NMR Spectrum of compound (*S*)-**2** (500 MHz, $CDCl_3$)

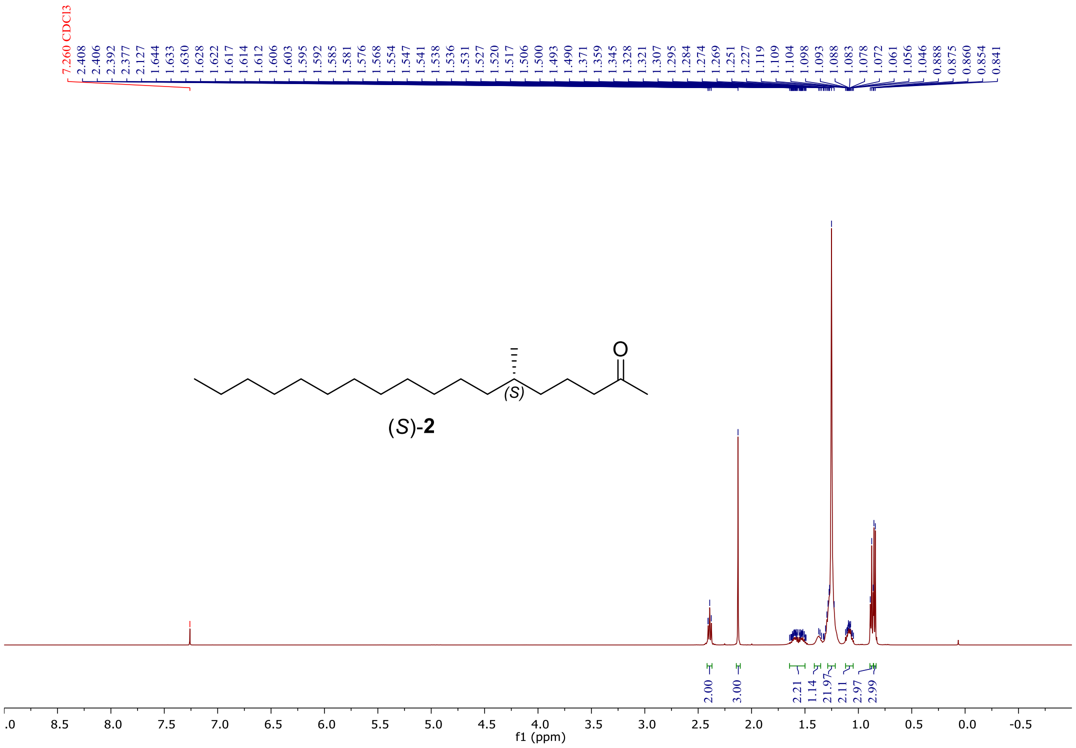

Figure S46. ^{13}C NMR Spectrum of compound (*S*)-**2** (125 MHz, $CDCl_3$)

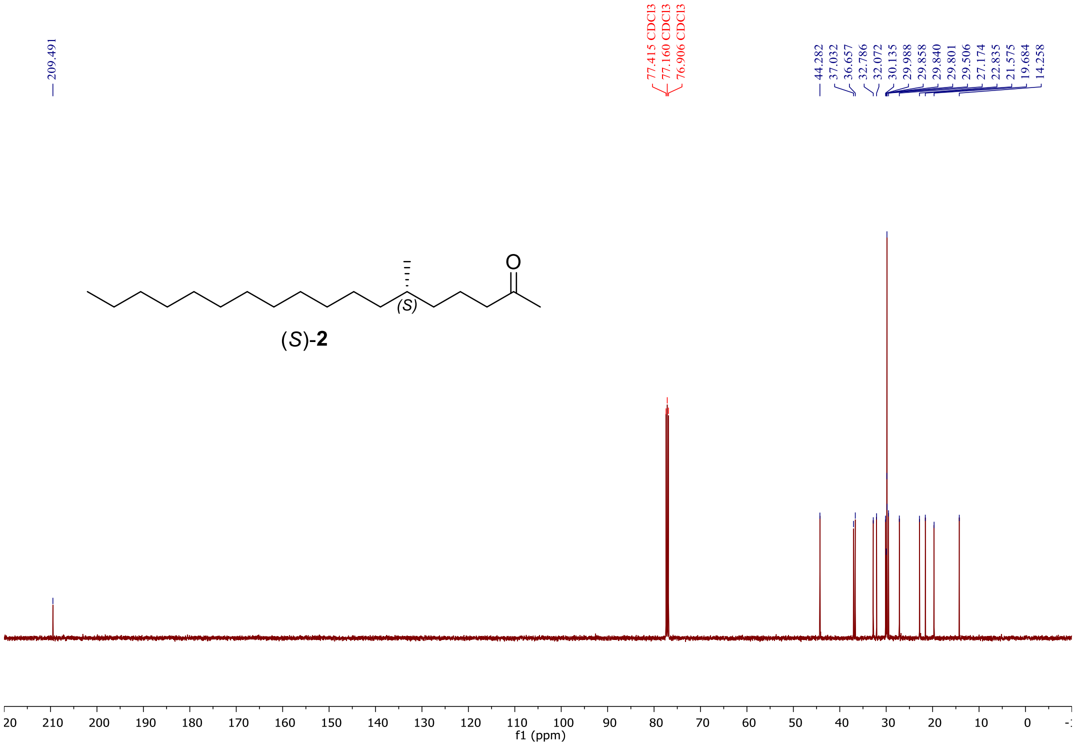

Figure S47. ^{1}H NMR Spectrum of compound (*R*)-**2** (500 MHz, $CDCl_3$)

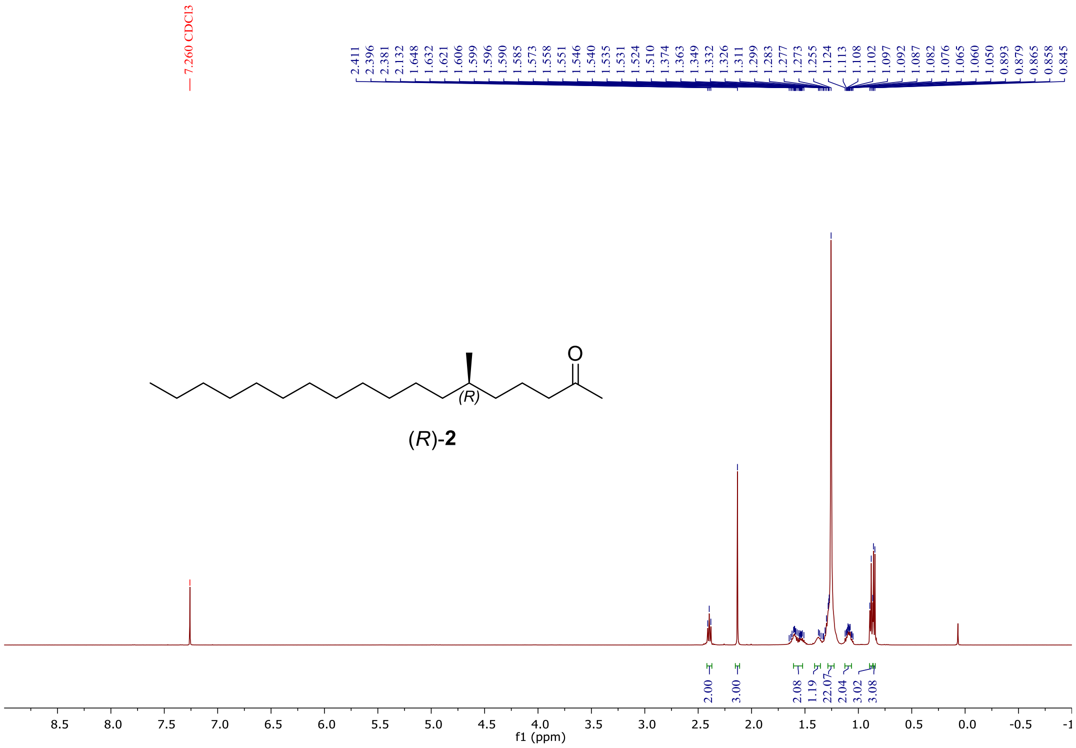

Figure S48. ^{13}C NMR Spectrum of compound (*R*)-**2** (125 MHz, $CDCl_3$)

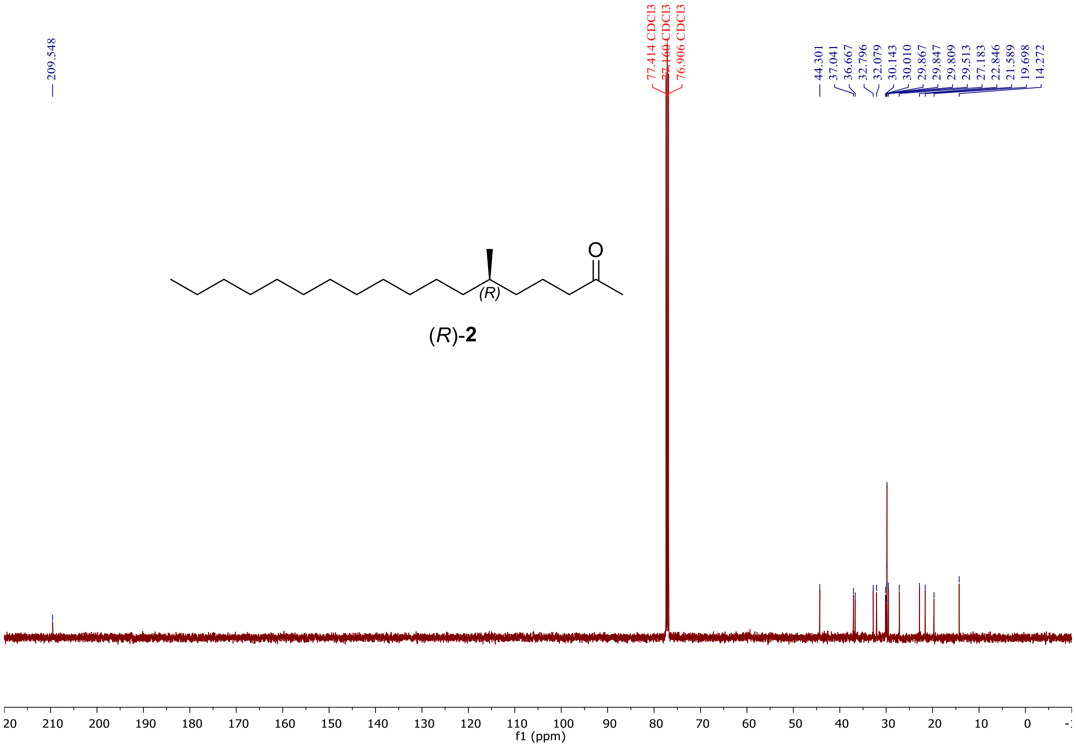

Figure S49. ^{1}H NMR Spectrum of compound **18** (500 MHz, $CDCl_3$)

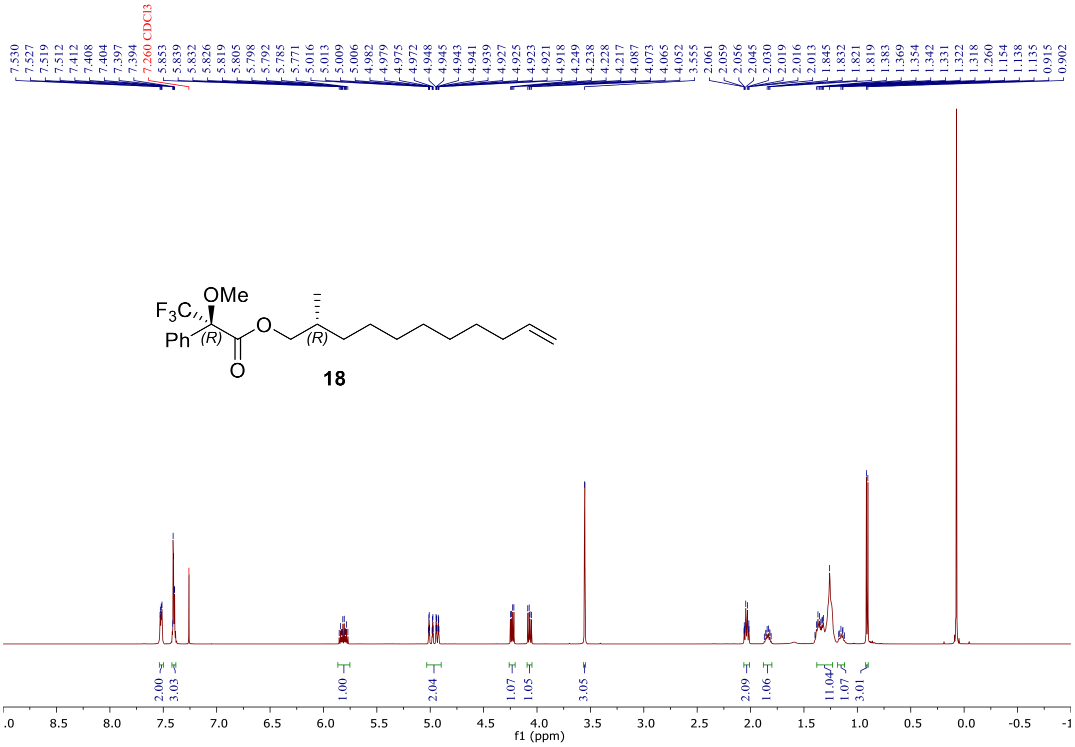

Figure S50. ^{1}H NMR Spectrum of compound **19** (500 MHz, $CDCl_3$)

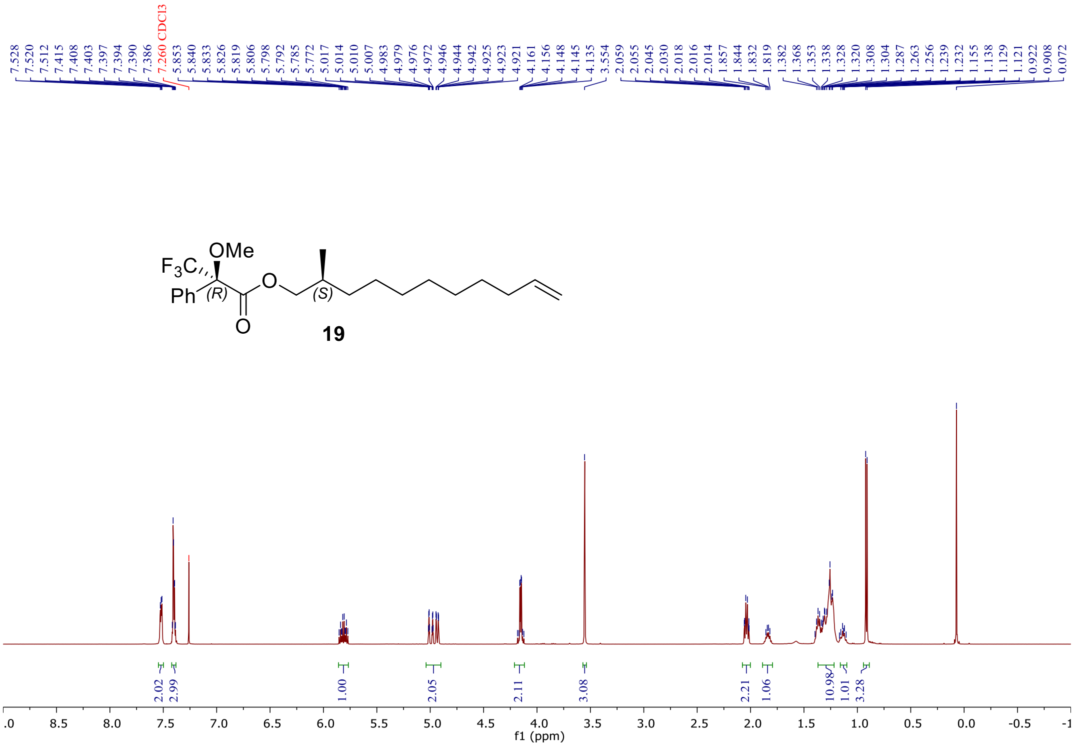

Figure S51. ^{1}H NMR Spectrum of compound **20** (500 MHz, $CDCl_3$)

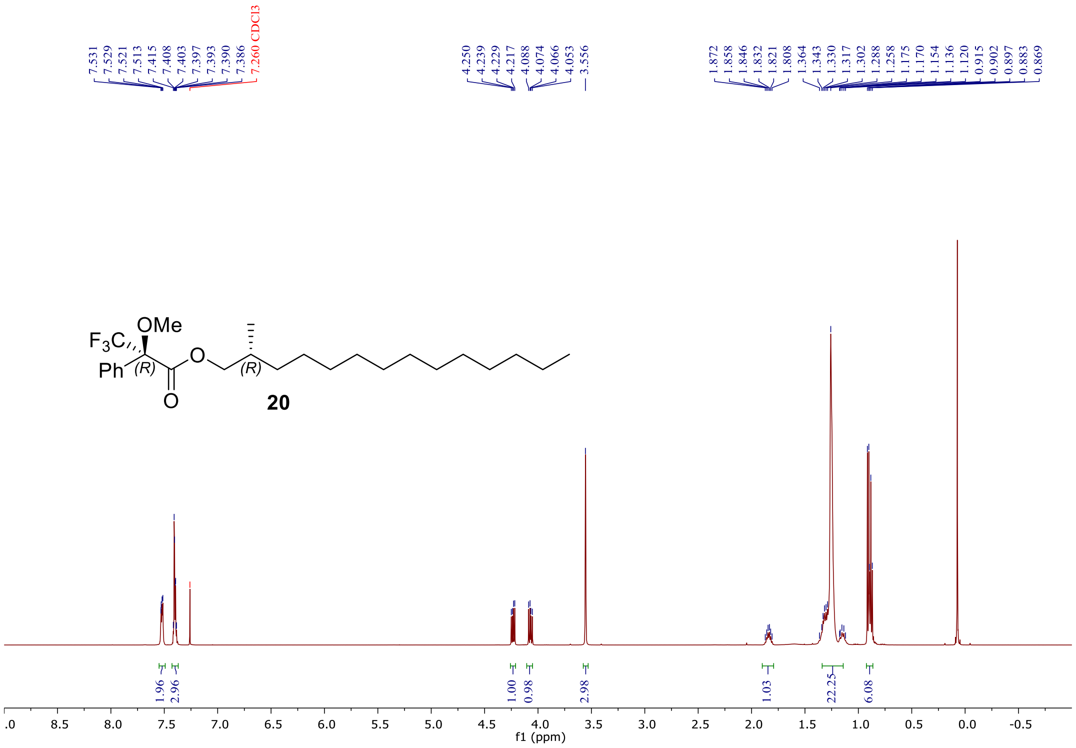

Figure S52. ^{1}H NMR Spectrum of compound **21** (500 MHz, $CDCl_3$)

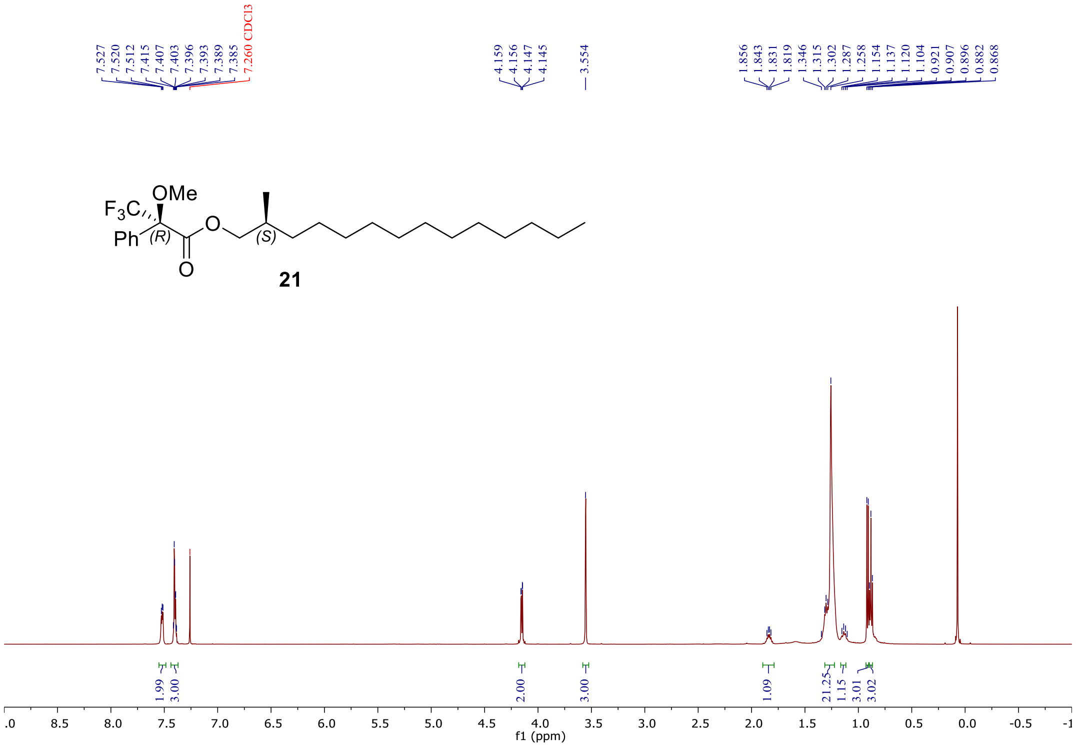

S4. Reference

1. Yuan, G.; Yang, Y.; Liu, J.; Bian, Q.; Wang, M.; Zhong, J. C., Synthesis of the enantiomers of 13 - methylheptacosane, the sex pheromone of pear psylla, *Cacopsylla pyricola*. *Chirality* **2021,** *33*, 274-280.
